# Metabolomic biomarkers of psychotic conversion in ultra-high-risk subjects: a pilot study

**DOI:** 10.1038/s41398-025-03679-8

**Published:** 2025-11-15

**Authors:** Maria Teresa Avella, Gildas Bertho, Nicolas Giraud, Oussama Kébir, Cédric Caradeuc, Javier Labad, Sergi Papiol, Thomas G. Schulze, Marie-Odile Krebs, Boris Chaumette, Ariel Frajerman

**Affiliations:** 1 Institute of Psychiatry and Neurosciences of Paris, INSERM UMR 1266, Pathophysiology of Psychiatric Diseases, Université Paris Cité, Paris, France; 2 Institute of Psychiatry, GDR 3557 of Psychiatry, GHU Paris, Paris, France; 3 Laboratoire de Chimie et Biochimie Pharmacologiques et Toxicologiques, UMR 8601 CNRS, Université Paris Cité, Paris, France; 4 MetaboParis-Santé, UMS BioMedTech Facilities - Inserm US36 | CNRS UAR2009 | Université Paris Cité, Paris, France; 5 Department of Mental Health and Addictions, Consorci Sanitari del Maresme, Mataro, Spain; 6 Translational Neuroscience Research Unit I3PT-INc-UAB, Institut de Innovacio Investigacio Parc Tauli (I3PT), Institut de Neurociències, Universitat Autònoma de Barcelona, Sabadell, Spain; 7 Centro de Investigacio Biomedica en Red deSalud Mental (CIBERSAM), Madrid, Spain; 8 Institute of Psychiatric Phenomics and Genomics (IPPG), LMU University Hospital, LMU Munich, Munich, BY Germany; 9 Max Planck Institute of Psychiatry, Munich, Germany; 10 Department of Psychiatry and Behavioral Sciences, Norton College of Medicine, SUNY Upstate Medical University, Syracuse, NY USA; 11 Department of Psychiatry and Behavioral Sciences, The Johns Hopkins University, Baltimore, MD USA; 12 PEPIT, GHU Paris Psychiatrie et Neurosciences, Paris, France; 13 Department of Psychiatry, McGill University, Montreal, Canada

**Keywords:** Predictive markers, Molecular neuroscience

## Abstract

Psychosis is a psychiatric condition that can become a chronic and severe psychiatric disorder affecting more than 1% of the population. The ultra-high risk (UHR) patients have a transition rate to psychosis of 25% after three years. We aimed to identify circulating metabolomic biomarkers for psychotic conversion in UHR patients using nuclear magnetic resonance (NMR) spectroscopy. We used samples from 35 UHR patients: 14 converters (UHR-C) and 21 non-converters (UHR-NC) at inclusion from the ICAAR cohort. Serum samples were analysed using the high-throughput screening IVDr NMR method. R and SIMCA were used for statistical analysis. Several lipoprotein parameters related to HDL and LDL metabolism were downregulated in UHR-C compared to UHR-NC at the time of inclusion. The 3 best lipoproteins to predict psychotic conversion at baseline were H4A1, H4FC, and L4FC (Area under the Curve (AUC) values were 0.81, 0.81, and 0.78, respectively). These lipoproteins were also negatively correlated with PANSS scores. Our study is the first to use NMR technology to identify biomarkers to predict the risk of psychotic transition in UHR subjects. This pilot study found lipoprotein parameters related to ApoA-1 and HDL-cholesterol (subclass 4) as potential biomarkers. These results need to be replicated on a larger sample. This study highlights the importance of the detailed analysis of circulant lipoproteins related to the brain using NMR technology in early psychosis to identify biomarkers of psychotic transitions and perhaps to better understand the physiopathology of psychosis.

## Introduction

Psychosis is a severe psychiatric condition occurring in several mental disorders, with a lifetime prevalence of psychosis ranging from 1.5–3.5% [1]. Because schizophrenia is the leading cause of psychosis, most studies focus on schizophrenia. The disease’s onset usually occurs between late adolescence and early adulthood. It could be preceded by a relatively non-specific period of symptoms, which led to the concept of at-risk mental state or ultra-high risk (UHR) [2]. UHR patients have a variable transition rate to psychosis of 15% within the first year from the diagnosis and 25% after three years [3]. A major aim for research is to identify biomarkers that predict psychotic transition and to find ways to prevent it [4]. Among biological markers, many studies using chromatography and mass spectrometry found lipid abnormalities linked to early psychosis. In UHR patients, lipid abnormalities were found before psychotic transition [5, 6]. In the First Episode of psychosis (FEP), abnormalities have been found in cholesterol lipoproteins (High-Density Lipoprotein (HDL) and Low-Density Lipoprotein (LDL)) and triglycerides [7] and in glycaemic control [8].

In addition, many CNS disorders are associated with disturbances of the plasma lipoprotein profile, and there is increasing evidence for the pathogenic and clinical relevance of these alterations [9].

Brain cholesterol and lipid homeostasis are mainly independent of plasma lipoproteins because the blood-brain barrier (BBB) restricts the transport of these molecules. ApoA-1 plays a major role in reverse cholesterol transport. In vitro studies have suggested apoA-I expression by brain endothelium and that plasma HDL (containing apoA-I) is transcytosis across the BBB [10]. In consequence, the implications of plasma lipoprotein metabolism in brain physiology and pathological states have been controversial.

Lipoproteins transport highly hydrophobic lipids (triglycerides, cholesterol) in blood, an aqueous fluid. There are 4 subclasses of lipoproteins: Chylomicrons, very low-density lipoproteins (VLDL), low-density lipoproteins (LDL), and high-density lipoproteins (HDL). Chylomicrons are synthesised by the intestine from triglycerides and cholesterol absorbed during a meal. Their main role is to transfer triglycerides through the blood to storage sites, adipose tissue, distributed throughout the body. VLDL are assembled by the liver from triglycerides, either newly synthesised or absorbed (from adipose tissue). VLDL transports triglycerides primarily to the muscles. LDLs are the major cholesterol transporters in the blood; they are synthesised by the liver and transport cholesterol to other cells in the body. HDLs transport cholesterol, much less than LDL, to the liver [11]. Inside each subclass (HDL, LDL, VLDL, chylomicron) are subclasses based on the size of the proteins. Still, standard methods (enzymatic) used in routine cannot discriminate and only give global results, whereas nuclear magnetic resonance spectroscopy (NMR) can discriminate 112 different lipoproteins [12].

Over the last few years, numerous studies in the general population have focused on using NMR to identify biomarkers that predict cardiovascular and metabolic events. For example, strong independent associations of diverse biomarkers, quantified using targeted NMR-based metabolomic profiling, including lipoprotein particle size and composition, amino acids and fatty acids, with risk of incident Type 2 Diabetes (T2D) were observed [13]. Recently, a team developed a Metabolic Score (MetSCORE) based on NMR to discriminate patients with Metabolic Syndrome from the general population, with an AUROC of 0.94 (95% CI 0.920–0.952, p < 0.001). Their MetSCORE was also able to identify patients at risk of metabolic syndrome [14].

In psychiatry, NMR has also already been recently investigated in Major Depressive Disorder (MDD). The study found differences in plasma between MDD patients with complete remission (N = 47) and healthy controls (N = 72), suggesting that metabolomic signatures might exist in the long term in MDD patients, with metabolic impacts on physical health [15]. Another study identified a specific metabolomic signature associated with a depression profile characterised by atypical, energy-related symptoms [16]. In bipolar disorder, differences between bipolar patients (N = 26) and controls (N = 50) on lipid metabolism-related molecules and some other metabolites [17] were found. There are very few studies on early psychosis and none about the psychotic transition.

This pilot study aimed to identify blood metabolomic biomarkers for psychotic conversion in UHR patients using NMR.

## Materials and methods

### Population

UHR patients came from the project “*Influence of Cannabis in Adolescents and Young Adults at Risk Mental State*” (ICAAR), promoted by Sainte-Anne Hospital (PHRC AOM 07 118, Principal Investigator MOK) between 2009 and 2014. The cohort was previously described [18]. Briefly, subjects (15–30 years old) were referred to a specialised outpatient clinic. The inclusion criteria were an alteration in global functioning (Social and Occupational Functioning Assessment Scale score <70) during the past year, associated with psychiatric symptoms and/or subjective cognitive complaints. Exclusion criteria for this cohort were manifest psychosis for more than one week, previous schizophrenia bipolar disorder, or pervasive developmental, current antipsychotic treatment ( > 100 mg/day Chlorpromazine equivalent) for more than 12 weeks; psychoactive substance dependence or abuse during the previous year and/or >5 years; severe somatic and neurological disorders; head injury; IQ lower than 70; and participants who were not native French speakers. 337 subjects were enrolled and interviewed at baseline using the Comprehensive Assessment for At-Risk Mental State (CAARMS, French version [19]) to determine if they reached the criteria for UHR or psychosis threshold. Subjects were followed for one year, and clinical evaluations were performed at inclusion (0 months), at 6 months (M6), and at 12 months (M12). Subjects who converted to psychosis during the follow-up were defined as “converters” and “non-converters”. The PANSS (Positive and Negative Symptoms Scales) was used to assess the severity of positive, negative and general symptoms [20].

### Ethics approval and consent to participate

This study was approved by the institutional ethics committee “Comité de protection des personnes, Ile-de—France III, Paris, France,” and written informed consent was obtained from all participants in accordance with the Declaration of Helsinki.

### Samples

Red blood cell (RBC) samples were stored at −80 °C in the Biological Resource Centre at GHU Paris Psychiatrie & Neurosciences. We collected available samples at the time of inclusion (M0). In the current analysis, we focused on UHR subjects and included 35 UHR patients: 14 converters (UHR-C) and 21 non-converters (UHR-NC), for whom samples were available and reliable clinical information was available.

### Sample preparation

Samples were thawed at room temperature for 30 min. The following steps were performed according to the Bruker IVDr NMR SOP as described elsewhere in detail [21]. In brief, 350 μL of Bruker Plasma Buffer and 350 μL of plasma were added into a 1.5 mL Eppendorf tube and gently shaken, and then 600 μL of the mixture was transferred into a 5 mm NMR tube for measurement.

### Spectral acquisition and quantification by ^1^H NMR spectroscopy

Serum samples were analysed using the high-throughput screening NMR platform ‘MetaboParis-Santé’ of the University Paris Cité (www.metaboparis.fr), which exploits the In Vitro Diagnostic for Research (IVDr) technique. This method relies on Standard Operating Procedures (SOPs) to prepare globally established biofluid samples. It shows various advantages, such as being non-destructive, fully automated, cost-effective and highly reproducible, such that the results can be repeated on another IVDr platform. More specifically, IVDr does not require operator expertise in NMR since it allows obtaining an automated quantitative report of the primary samples’ metabolites. Furthermore, it makes it possible to compare the results of different research groups worldwide [21].

Plasma-EDTA samples were analysed on a 600 MHz IVDr NMR system (Bruker NEO console) operated with double resonance (BBI) room temperature 5 mm probe with z-gradients at 310 K. Standard nuclear Overhauser spectroscopy experiment (1D-NOESY) used in metabolomics was run over 4 min, to quantify 38 polar metabolites and 112 lipoproteins parameters by automated Bruker IVDr quantification of small molecule metabolites (B.I.QUANT-PS^TM^) and Bruker IVDr Lipoprotein Subclass Analysis (B.I.LISA^TM^), respectively [22].

### Statistical analysis

T-tests for the continuous variables (age, BMI, PANSS scores) and the chi-squared test for categorical variables (sex, antipsychotic) were performed with R (version 4.3.0) [23] using the statistical analysis *gtsummary* package. Multivariate analysis was performed with SIMCA 17 [24]. We first conducted Principal Component Analysis (PCA) to check the overall quality of samples and NMR spectroscopy measurements. Subsequently, we used the supervised method of Orthogonal Partial Least Squares Discrimination Analysis (OPLS-DA) to investigate variance correlated to conversational status. Univariate analysis was performed with the online platform MetaboAnalyst 6.0 [25]. We used a parametric t-test to compare the serum concentration of metabolites and lipoprotein parameters between UHR-C and UHR-NC. We decided on a significance threshold value of 0.05 for p-values. To evaluate the performance of our classification model, we plot Receiver Operating Characteristic (ROC) curves with MetaboAnalyst 6.0. The AUC (Area Under the Curve) was calculated to measure the model’s discriminative ability. Additionally, a p-value was computed to test if the AUC is significantly different from 0.5, indicating that our model performs better than random chance. A significance level of 0.05 for the p-value was chosen. A correlation analysis (Pearson) between lipoprotein parameters that significantly predicted conversion to psychosis and the severity of clinical symptoms was measured by PANSS with R packages *corrplot* and *Hmisc*.

Analysis for ROC curves and correlations were also performed on men and women separately.

## Results

### Socio-demographic features

The cohort comprised more males than females (21 vs. 14, respectively), with a mean ± standard deviation age of 21.5 ± 3.55 years and a mean BMI of 22.69 ± 3.99 kg/m^2^. At inclusion, there was no difference between future converters (C) and non-converters (NC) for age, gender, or BMI (Table 1).

**Table 1:** Clinical characteristics of the population at the time of inclusion.

Characteristic	UHR-C, N = 14^*1*^	UHR-NC, N = 21^*1*^	p-value^*2*^
**Sex**			>0.9
Women	6 (43%)	9 (43%)	
Men	8 (57%)	12 (57%)	
**Age**	20.71 ± 2.95	21.33 ± 4.08	0.6
**BMI**	22.31 ± 4.17	22.99 ± 4.14	0.6
**Antipsychotic**	0 (0%)	4 (19%)	<0.001
**PANSS Total**	80.00 ± 18.88	57.10 ± 12.71	<0.001
**PANSS General**	44.07 ± 10.34	34.95 ± 8.02	0.010
**PANSS Positive**	18.79 ± 5.12	9.86 ± 3.24	<0.001
**PANSS Negative**	17.14 ± 6.83	12.29 ± 4.82	0.031

1 n (%); Mean ± SD.

2 Pearson’s Chi-squared test; Welch Two Sample t-test.

*UHR-C* ultra-high risk patient converters, *UHR-NC* ultra-high risk patient non-converters, *BMI* body mass Index, *PANSS* positive and negative symptoms scales.

### Analyses at inclusion

**Fig. 1:**
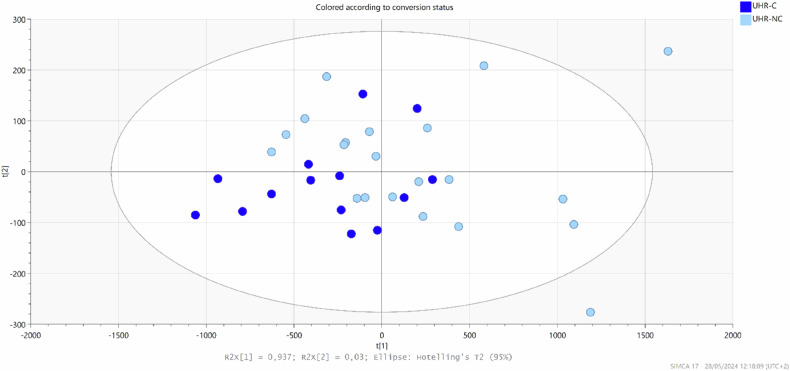
Principal Component Analysis (PCA) of UHR (Ultra-High Risk) subjects at inclusion (M0). Blue: UHR-C, Light blue: UHR-NC.

Principal component analysis (PCA) (Fig. 1) and orthogonal partial least square discrimination (OPLS-DA) (Supplementary Fig. 1) conducted on subjects at baseline did not highlight differences between UHR-C and UHR-NC.

**Fig. 2:**
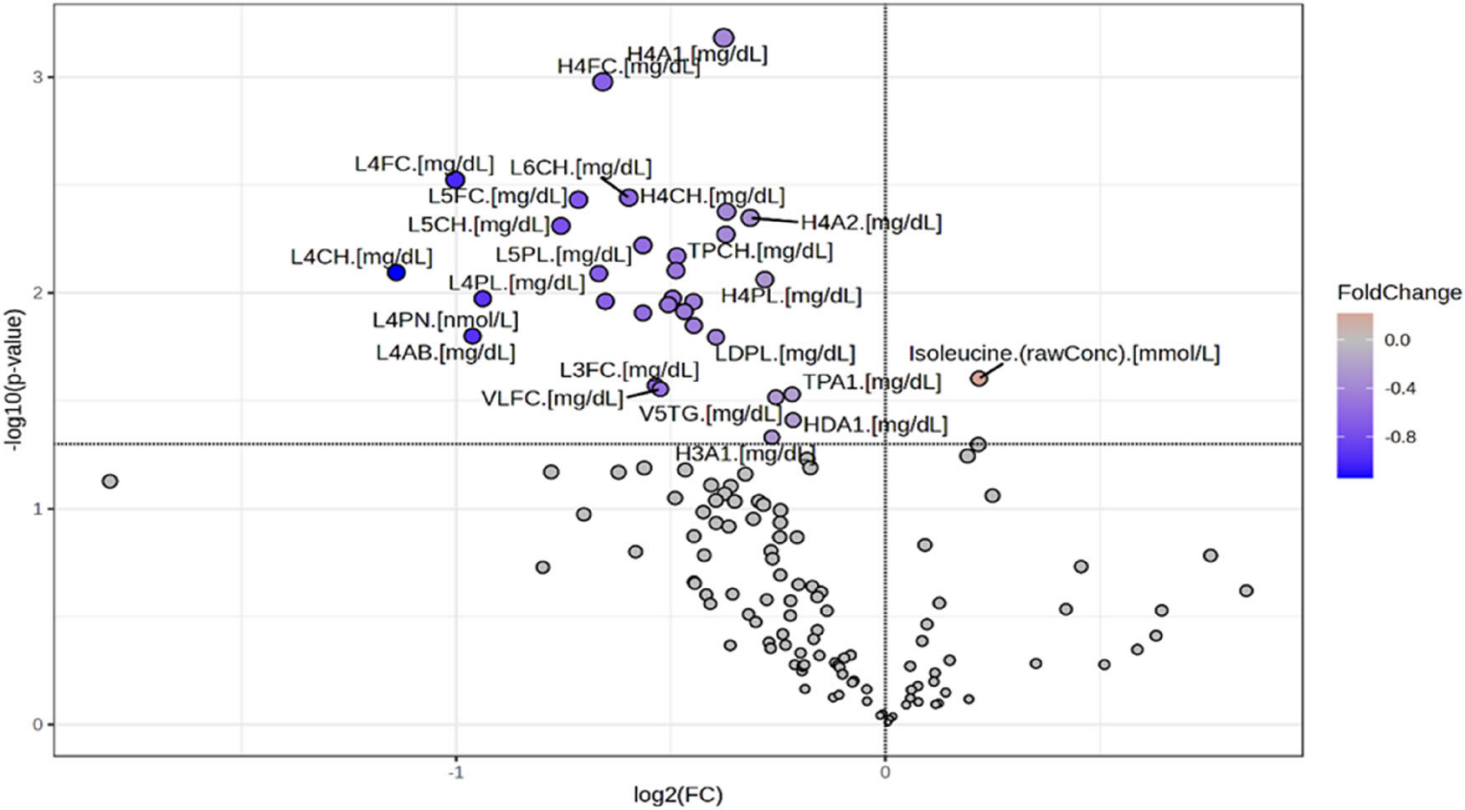
Volcano Plot of UHR-C (Ultra-High Risk-Converter) /UHR-NC (Ultra-High Risk-Non Converter) at inclusion (M0).

Several lipoprotein parameters related to HDL and LDL metabolism were downregulated in UHR-C compared to UHR-NC at M0 (Fig.2), with the most relevant being H4A1 (Apo-A1 in HDL-4) and H4FC (Free cholesterol in HDL-4) (Table 2). However, this finding did not remain significant after the FDR correction (Supplementary Table 1).

**Table 2:** Significant differences in metabolites and lipoprotein parameters between UHR-C and UHR-NC.

	FC	log2FC	p-value
H4A1 [mg/dL]	0.77012	−0.37685	0.00065807
H4FC [mg/dL]	0.63355	−0.65847	0.0010514
L4FC [mg/dL]	0.4992	−1.0023	0.0029945
L6CH [mg/dL]	0.66078	−0.59776	0.0036255
L5FC [mg/dL]	0.60908	−0.71529	0.0037058
H4CH [mg/dL]	0.77371	−0.37014	0.0042033
H4A2 [mg/dL]	0.80395	−0.31483	0.0044975
L5CH [mg/dL]	0.5921	−0.75608	0.004899
TPCH [mg/dL]	0.77281	−0.37181	0.0053653
L6AB [mg/dL]	0.67602	−0.56485	0.0060256
L6PN [nmol/L]	0.67602	−0.56486	0.0060285
TPAB [mg/dL]	0.71406	−0.48589	0.0067591
TBPN [nmol/L]	0.71406	−0.48589	0.0067605
LDFC [mg/dL]	0.71305	−0.48792	0.0078898
L4CH [mg/dL]	0.45393	−1.1395	0.0080557
L5PL [mg/dL]	0.62947	−0.66778	0.0081426
H4PL [mg/dL]	0.82307	−0.28091	0.0086849
LDCH [mg/dL]	0.70934	−0.49546	0.010606
L4PL [mg/dL]	0.52194	−0.93805	0.010656
L5AB [mg/dL]	0.63619	−0.65247	0.010958
L5PN [nmol/L]	0.63618	−0.65249	0.01097
L6PL [mg/dL]	0.73343	−0.44726	0.01099
L6FC [mg/dL]	0.70428	−0.50577	0.011363
LDPN [nmol/L]	0.72282	−0.46829	0.012228
LDAB [mg/dL]	0.72283	−0.46828	0.012229
H3FC [mg/dL]	0.67609	−0.56472	0.012409
HDFC [mg/dL]	0.73393	−0.44628	0.014205
L4PN [nmol/L]	0.51328	−0.96219	0.015869
L4AB [mg/dL]	0.51342	−0.96178	0.015894
LDPL [mg/dL]	0.76068	−0.39463	0.01608
Isoleucine (rawConc) [mmol/L]	1.1634	0.21839	0.02499
L3FC [mg/dL]	0.68977	−0.5358	0.026898
VLFC [mg/dL]	0.69529	−0.52432	0.02799
TPA1 [mg/dL]	0.8602	−0.21725	0.029576
V5TG [mg/dL]	0.83785	−0.25524	0.030538
HDA1 [mg/dL]	0.86151	−0.21505	0.038848
H3A1 [mg/dL]	0.83246	−0.26454	0.046589

*UHR-C* ultra-high risk patient converters, *UHR-NC* ultra-high risk patient non-converters, *FC* fold change.

There were also significant differences between women and men, but these findings did not remain significant after the FDR correction (Supplementary Table 3).

We did the same analysis after exclusion of the 4 subjects using antipsychotics and found similar results (Supplementary Table 4).

### Prediction of psychotic transition

**Fig. 3:**
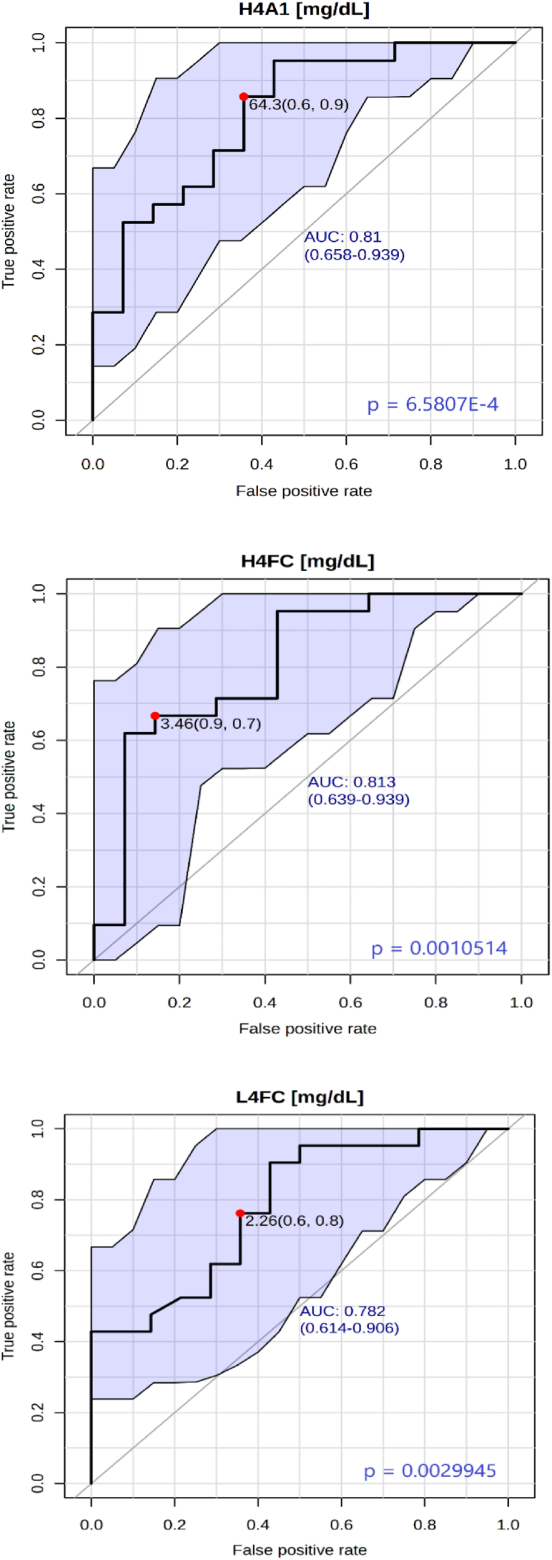
ROC curves of lipoprotein parameters for all subjects at inclusion.

Several lipoprotein parameters showed an excellent discrimination capacity between UHR-C and UHR-NC at baseline (Supplementary Table 2). The 3 best biomarkers (most discriminants) validated using ROC curve analysis were H4A1 (Apo-A1 in HDL-4; area under the curve (AUC) = 0.81), H4FC (Free Cholesterol in HDL-4; AUC = 0.81), and L4FC (Free Cholesterol in LDL-4; AUC = 0.78) (Fig.3). We observed sex differences in the dataset. The 3 best lipoproteins for males were H4A1, H4FC and VLPL (Phospholipids in VLDL) with AUC values of 0.89, 0.92 and 0.85, respectively (Supplementary Fig. 2). For females, the three best parameters were LDFC (Free Cholesterol in LDL), TPCH (Total Cholesterol), and LDCH (LDL Cholesterol), with AUC values of 0.93, 0.89, and 0.89, respectively (Supplementary Fig. 3).

We did the same analysis after exclusion of the 4 subjects using antipsychotics, and the 3 ROC curves remained significant (Supplementary Figs. 9–11).

### Correlations between PANSS scores and lipoprotein parameters

**Fig. 4:**
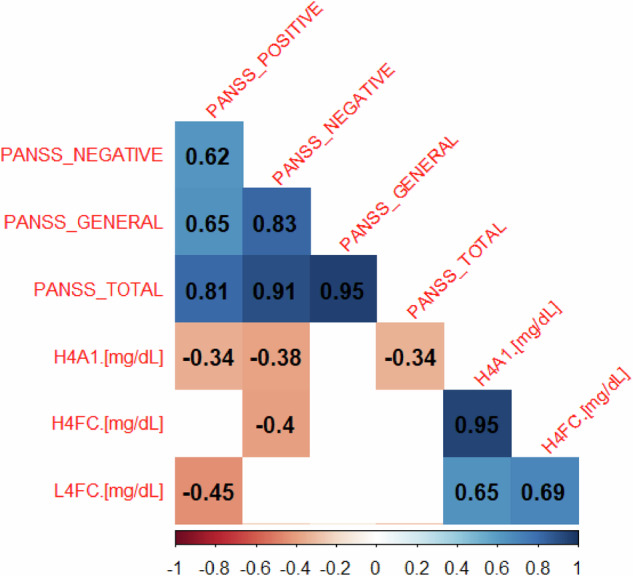
Correlation plot of Positive and Negative Syndrome Scale (PANSS) Scores and lipoprotein parameters for all subjects at inclusion.

We studied the correlation between the 3 best lipoproteins and symptoms using the PANSS scores at inclusion. For all subjects, H4A1 and H4FC were negatively correlated with negative symptoms, H4A1 and L4FC were negatively correlated with positive symptoms, and only H4A1 was negatively correlated with total PANSS scores (Fig.4). For men (n = 21), only VLPL was negatively correlated with positive and total PANSS scores (Supplementary Fig. 5). For women (n = 14), L4FC, LDFC, TPCH, and LDCH were negatively correlated with positive symptoms, and L4FC was also negatively correlated with total PANSS scores (Supplementary Fig. 4).

We did the same analysis after exclusion of the 4 subjects using antipsychotics and found stronger correlations (Supplementary figs. 6–8).

## Discussion

In this study, NMR analysis allows us to identify several metabolomic biomarkers that could predict transition to psychosis in UHR subjects: H4A1, H4FC, and L4FC. The concentrations of these 3 lipoproteins were also correlated with PANSS scores.

To our knowledge, this is the first report of NMR analysis in UHR regarding conversion to psychosis.

Previous studies found sex-specific associations between cholesterol and clinical symptoms. Some researchers observed a female-specific association between HDL cholesterol levels and negative symptoms, which remained significant after adjusting for BMI [26]. Another study in women with schizophrenia found significantly lower values of LDL cholesterol in the group of female patients with aggressive behaviour compared to nonaggressive patients [27].

### NMR studies in early psychosis

There is only one study on NMR in early psychosis. A Spanish study included 67 healthy controls (HCs), 58 UHR, 110 First Episodes of psychosis (FEP) (N = 110) and 53 early psychosis diagnoses with more than 2 episodes (critical period (CP)). They found a gradual increase in the Glyc levels from HCs to CP patients; this increase was statistically significant for GlycA (CP vs HC). They also observed a progressive and significant proatherogenic 1H NMR lipoprotein profile across stages of psychosis (ARMS and CP vs HC). However, they did not study the psychotic transition risk [28]. Another study included 72 HC, 52 Hepatitis B virus (HBV) infection, 37 schizophrenia patients (SZ), and 41 SZ + HBV patients. The SZ group exhibited increased levels of betaine, choline, citrate, lactate, pyruvate, threonine, and tyrosine, along with decreased levels of creatinine, formate, glucose, glutamine, glycerol, and N-acetyl-glycoprotein signals compared to HC [29].

### Lipids and lipoproteins abnormalities in Psychosis

The findings of lipoprotein differences between converters and non-converters raise questions about the significance of these abnormalities. Previous studies employed standard measures and were unable to differentiate between the various types of lipoproteins. A meta-analysis found that FEP patients had lower total cholesterol (TC) and LDL cholesterol levels, but higher triglyceride (TG) levels, compared with healthy subjects. There was no difference in HDL cholesterol [30]. However, in a more recent meta-analysis, mean CT and mean HDL levels were reduced, and mean TG was increased in FEP patients compared with controls, and there was no difference in mean LDL level [7]. Other studies focused on lipoproteins and symptoms. A study on 132 FEP patients found that an increase in HDL level during one year of antipsychotic treatment was associated with a reduction in PANSS-negative symptoms, and this association persisted after adjusting for BMI [31].

Following the membrane hypothesis of schizophrenia [32], lipid abnormalities were previously reported in schizophrenia and in drug-naïve FEP patients [7]. Most studies focus on fatty acids (FA). Subgroups of schizophrenic patients were identified based on membrane lipids [33, 34]. In UHR subjects, we found that membrane lipids (polyunsaturated fatty acids (PUFA), phospholipids, and sterols) could help to predict the risk of conversion to psychosis [6]. This could have therapeutic consequences, such as omega-3 supplementation recommendations for schizophrenic patients [35]. A recent network meta-analysis concluded the superior effects of omega-3 polyunsaturated fatty acids in preventing the transition to psychosis in UHR subjects [36]. However, omega-3 is involved in many biological pathways (inflammation, oxidative stress, monocarbon, …), and it is difficult to identify which pathway is primarily disturbed [37].

### Lipids and Lipoproteins in other neuropsychiatric disorders

The APOE gene codes for an apolipoprotein [38]. A meta-analysis suggested that APOE4 was associated with worse severity of hallucinations and delusions in late adulthood among schizophrenic patients [39]. Although no relation exists between plasma and brain apoE levels, a strong correlation was found between HDL-cholesterol and apo-A1 in serum and in Cerebrospinal Fluid (CSF) lipoproteins (which are HDL-like particles). This scenario could contribute to the risk of cognitive impairment and AD conferred by low plasma HDL-cholesterol and Apo-A1 levels. On the one hand, these deficiencies could be linked to an increased systemic inflammatory and oxidative status and the promotion of atherogenesis. On the other hand, low HDL and Apo-A1 levels would provide less neurotrophic and immunosuppressive abilities to the brain [40]. Lipoprotein remodelling in the periphery has many component processes and is highly complex. Perhaps the best peripheral model for the proposed process of lipoprotein biogenesis in the brain is an adaptation of ApoA1-HDL-mediated reverse cholesterol transport (RCT). Recently, an NMR study found that serum HDL-4 parameters and various triglycerides correlate positively with AD pathology [41].

Therefore, there is an increasing interest in lipids in psychiatry. There is now much data on the use of lipids as biomarkers [42], but less on physiopathology. Recently, two articles suggested that lipids could be the missing pathophysiologic link to better understand psychiatric diseases and the action of psychiatric treatments [43, 44].

### Antipsychotics and cholesterol metabolism

Many studies have found an impact of antipsychotics on cholesterol metabolism. Haloperidol, Ziprasidone, and Risperidone inhibit cholesterol biosynthesis by affecting Δ 7—reductase, Δ 8,7—isomerase, and Δ 14—reductase activities, whereas clozapine mainly affected Δ 24—reductase and Δ 8,7—isomerase activities [45]. A transcriptomic in vitro study on neuronal cells found a profound effect of clozapine on cholesterol metabolism [46].

Several studies found decreased HDL cholesterol levels in patients treated with atypical antipsychotics (APA). In vitro, many antipsychotics (haloperidol, pimozide, aripiprazole,

clozapine, quetiapine, olanzapine, risperidone, and ziprasidone) inhibit enzymes in the

final steps of cholesterol biosynthesis, reducing cellular cholesterol content and inducing the accumulation of sterol intermediates. The impact of antipsychotics on cholesterol could be linked to their actions on the sterol regulatory-element binding proteins (SREBPs) pathway, but also the insulin-induced genes (INSIGs) or the AMP-activated protein kinase (AMPK) pathway [47]. Another review suggested that the impact of antipsychotics on lipids is related to the type of receptor they linked: higher occupancy of H1 histaminergic and M1 as well as M3 cholinergic receptors was associated with higher circulating total cholesterol and LDL-C, while M3 cholinergic occupancy negatively impacted HDL-C. Furthermore, they had different actions regarding the organ [48]. Antipsychotic action on lipid metabolism could partially explain their efficiency on psychosis and is a lead for new treatments. In our study, UHR patients (except 4 in the NC group) had no antipsychotics at inclusion, so differences in lipoprotein parameters between UHR-NC and UHR-C were not explained by treatment differences in antipsychotic drugs.

### Limitations and strengths

Our study had some limitations. First, this is an exploratory study with a low number of subjects, which explains why we did not adjust the p-value in Table 2. However, we added the adjusted p-value in the Supplementary Tables. Additionally, the correlations for all subjects were not significant when divided by sex. Second, we have a higher proportion of converters in our UHR population sample than in real life because of the proportion in the remaining samples. Third, not all parameters were implemented in the NMR device at the time of analysis, so we do not have the Glyc A parameter to compare with the previous NMR study on early psychosis. Finally, we did not have a healthy control group to compare.

However, the study also had strengths: UHR patients (except 4) had no antipsychotics at inclusion, eliminating an important bias. UHR-NC and UHR-C were well-matched for age, sex, and BMI, thereby avoiding differences driven by these potential confounders. There was a 12-month longitudinal follow-up to determine the converters.

## Conclusion

Our study is the first study using NMR technology to identify blood biomarkers to predict the risk of conversion to psychosis in UHR subjects. Namely, this pilot study found lipoprotein parameters related to apolipoprotein A1 (Apo-A1) and one of the larger-size HDL (subclass 4) as potential predictive biomarkers. Interestingly, such alterations in the levels of HLD-cholesterol and Apo-A1 were associated with many brain diseases. Whereas these results need replication in larger samples, it is in line with previous studies highlighting the importance of lipids and lipoproteins in psychiatry and psychosis, with differences regarding sex. This study further supports utilising NMR technology in early psychosis to identify circulating biomarkers in psychosis onset and potentially gain deeper insights into the pathophysiology of psychosis.

## Supplementary information

Supplementary materials

## Data Availability

The biological data analyzed during this study are available upon reasonable request from the authors.
