# Supplementary material for: Metabolomic biomarkers of psychotic conversion in ultra-high-risk subjects: a pilot study
Source: Transl Psychiatry. 2025 Nov 15;15:503. doi: 10.1038/s41398-025-03679-8 (PMC12644573; doi:10.1038/s41398-025-03679-8)
Supplement: Supplementary file 1 — Supplementary materials [file 41398_2025_3679_MOESM1_ESM.docx]

Supplementary materials

**Supplementary Figure 1: Orthogonal Partial Least Squares Discriminant Analysis (OPLS-DA) of UHR subjects at baseline.**

**Blue: UHR-C, Light blue: UHR-NC**


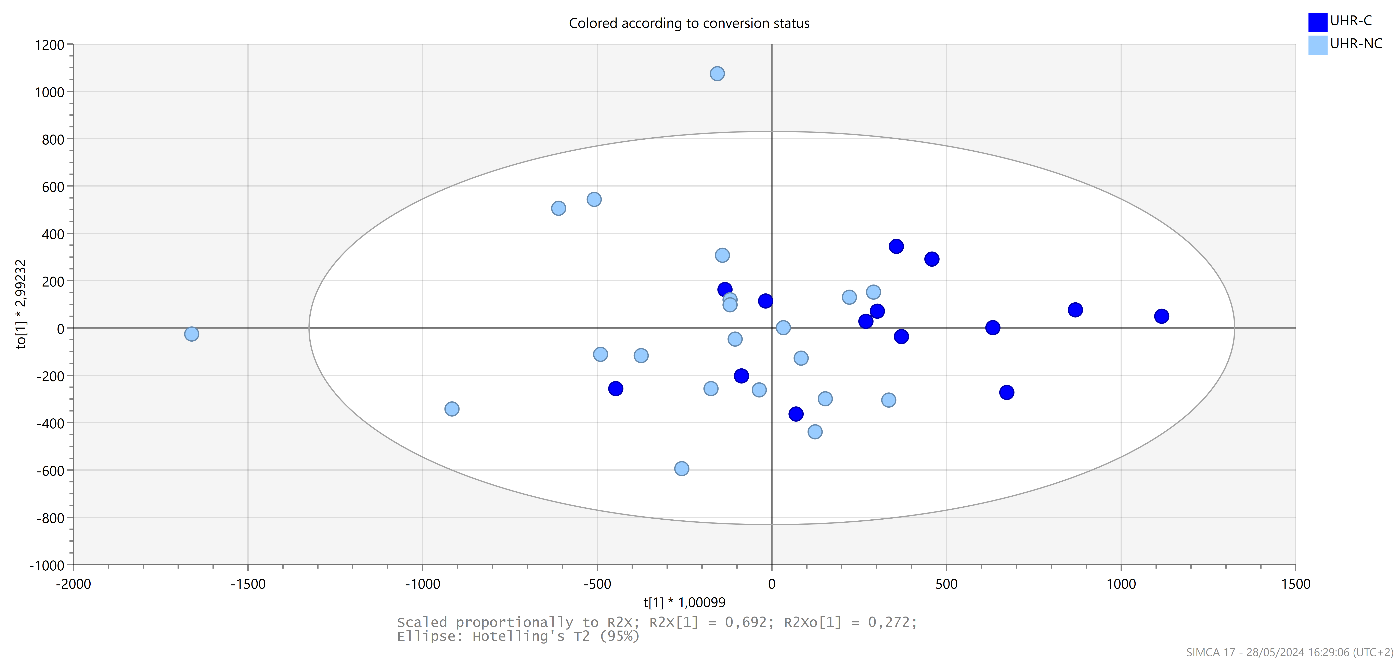


**Supplementary Figure 2: ROC curves for the 3 best parameters in males**


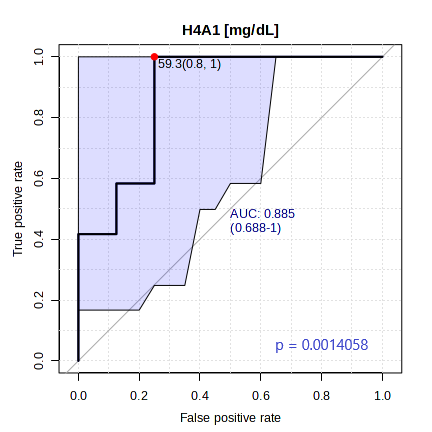


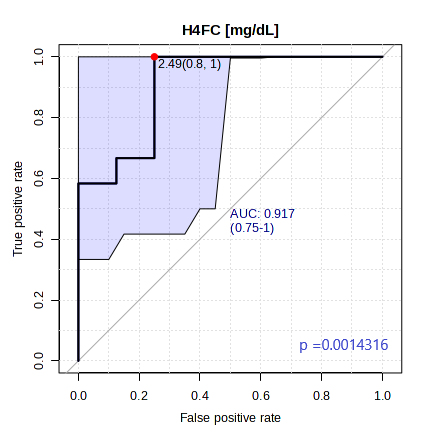


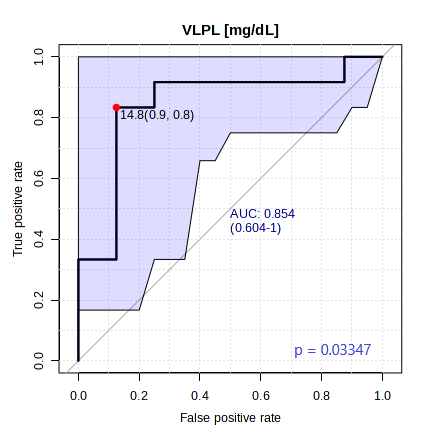


**Supplementary Figure 3: ROC curves for the 3 best parameters in females**


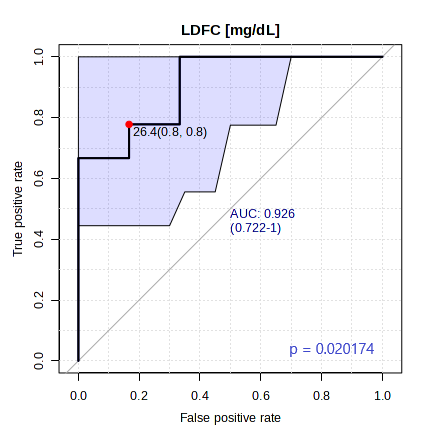


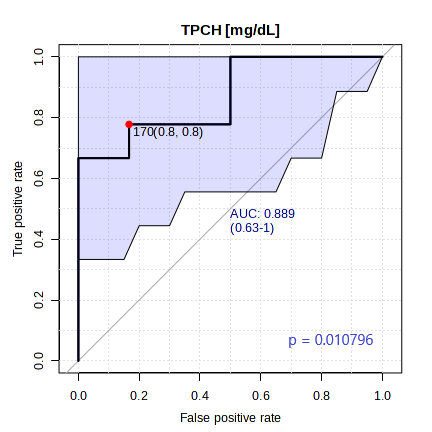


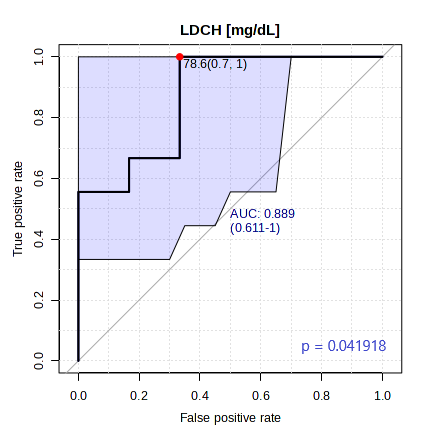


**Supplementary Figure 4: Correlation matrix of Positive and Negative Syndrome Scale (PANSS) Scores and lipoprotein parameters at inclusion for Women**

**
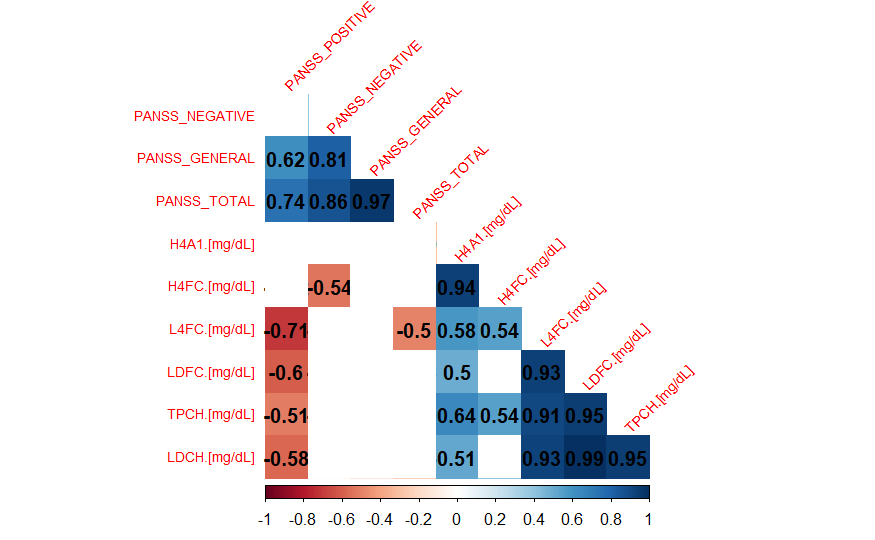
**

**Supplementary Figure 5: Correlation matrix of Positive and Negative Syndrome Scale (PANSS) Scores and lipoprotein parameters at inclusion for Men**


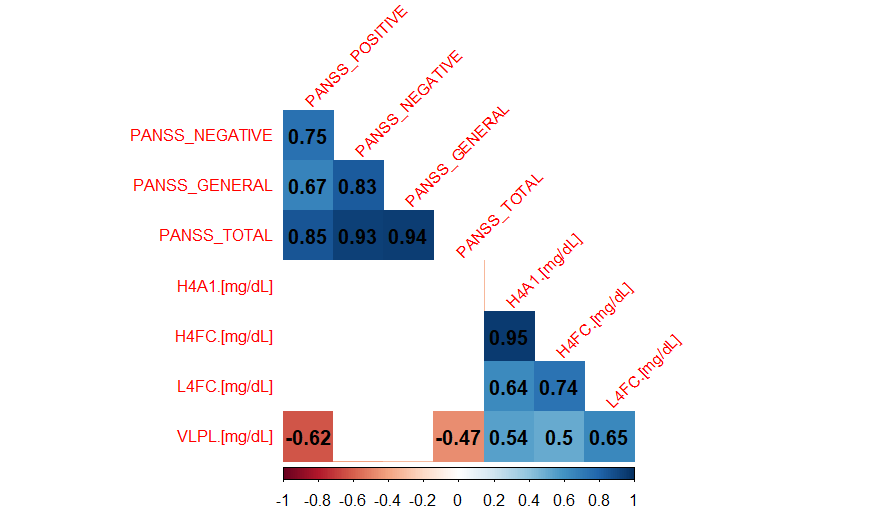


**Supplementary Figure 6: Correlation plot of Positive and Negative Syndrome Scale (PANSS) Scores and lipoprotein parameters for all subjects at inclusion after exclusion of subjects with antipsychotics**

**
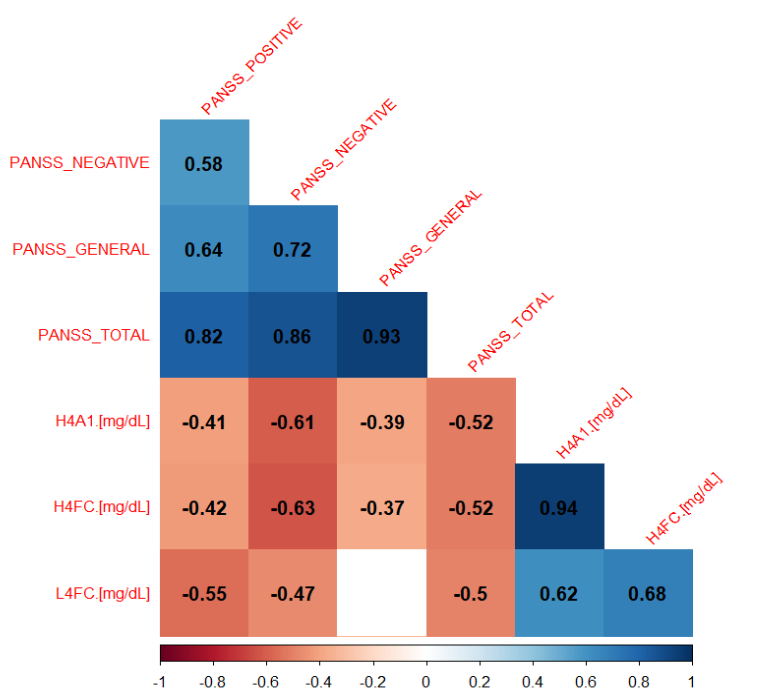
**

**Supplementary Figure 7: Correlation matrix of Positive and Negative Syndrome Scale (PANSS) Scores and lipoprotein parameters at inclusion for Women after exclusion of subjects with antipsychotics**

**
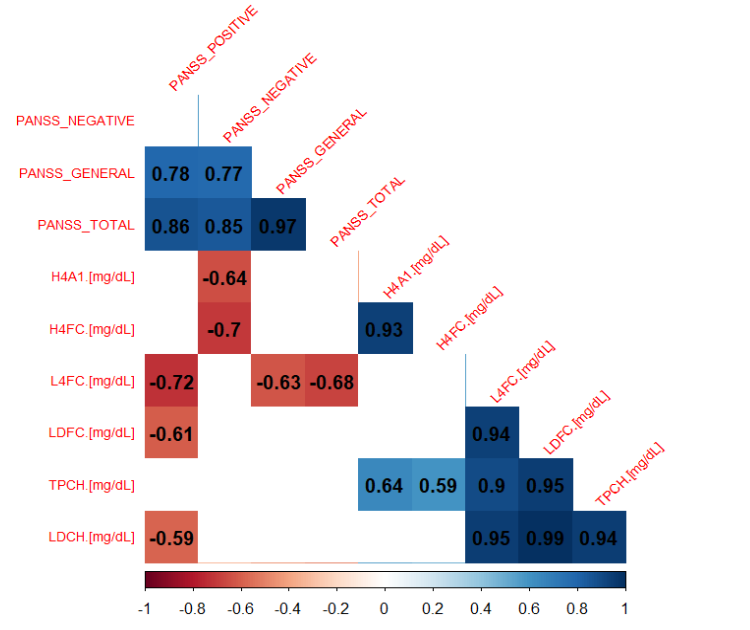
**

**Supplementary Figure 8: Correlation matrix of Positive and Negative Syndrome Scale (PANSS) Scores and lipoprotein parameters at inclusion for Men after exclusion of subjects with antipsychotics**


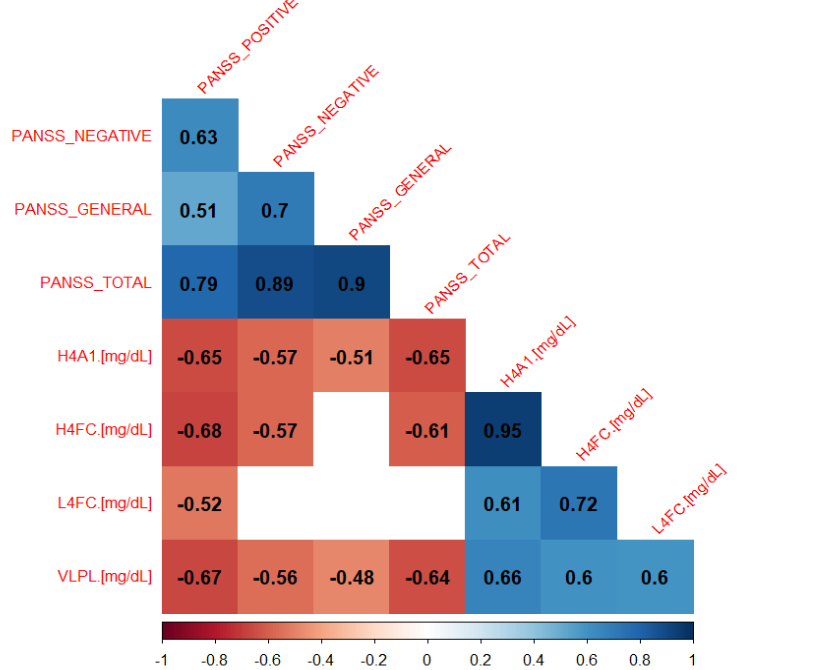


**Supplementary Figure 9:** **ROC curves for the 3 parameters after exclusion of the 4 subjects with antipsychotics**


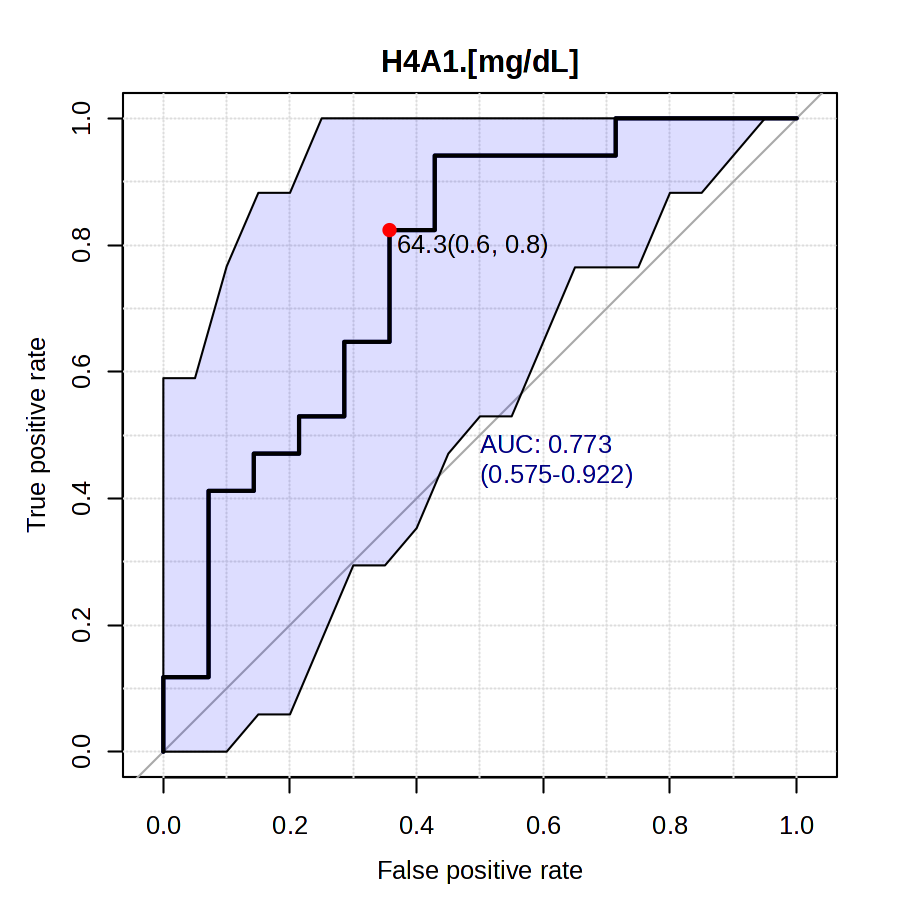


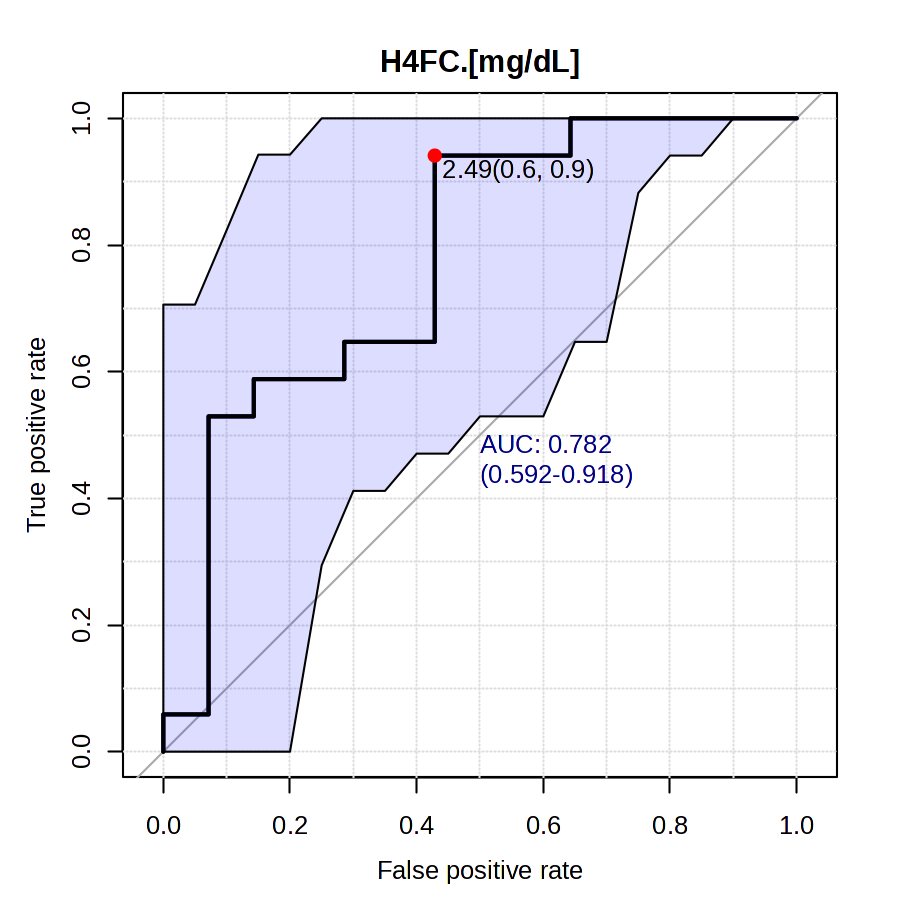


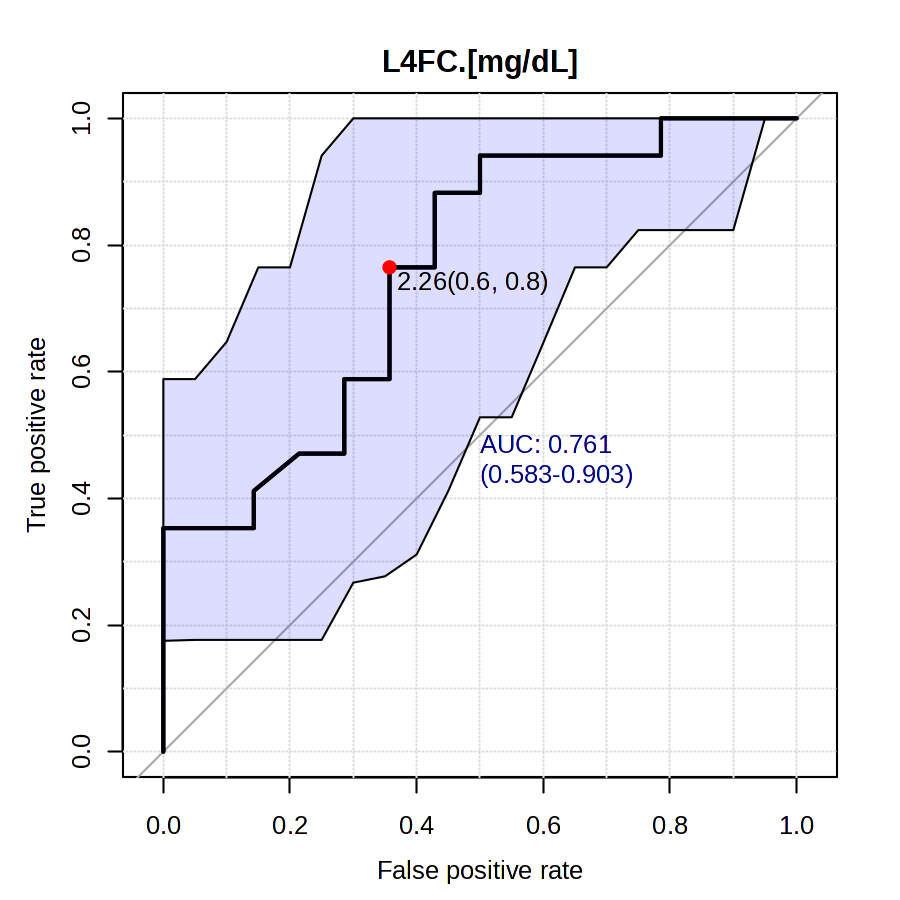


**Supplementary Figure 10: ROC curves for the 3 parameters for males after exclusion of the subjects with antipsychotics**


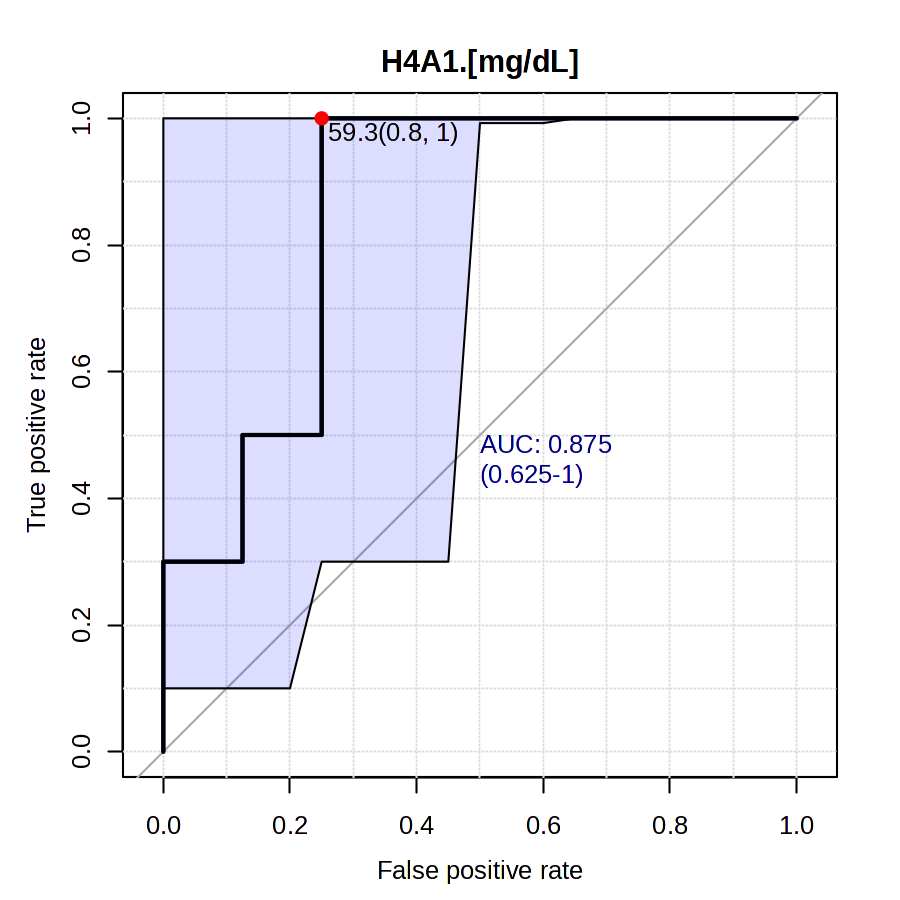


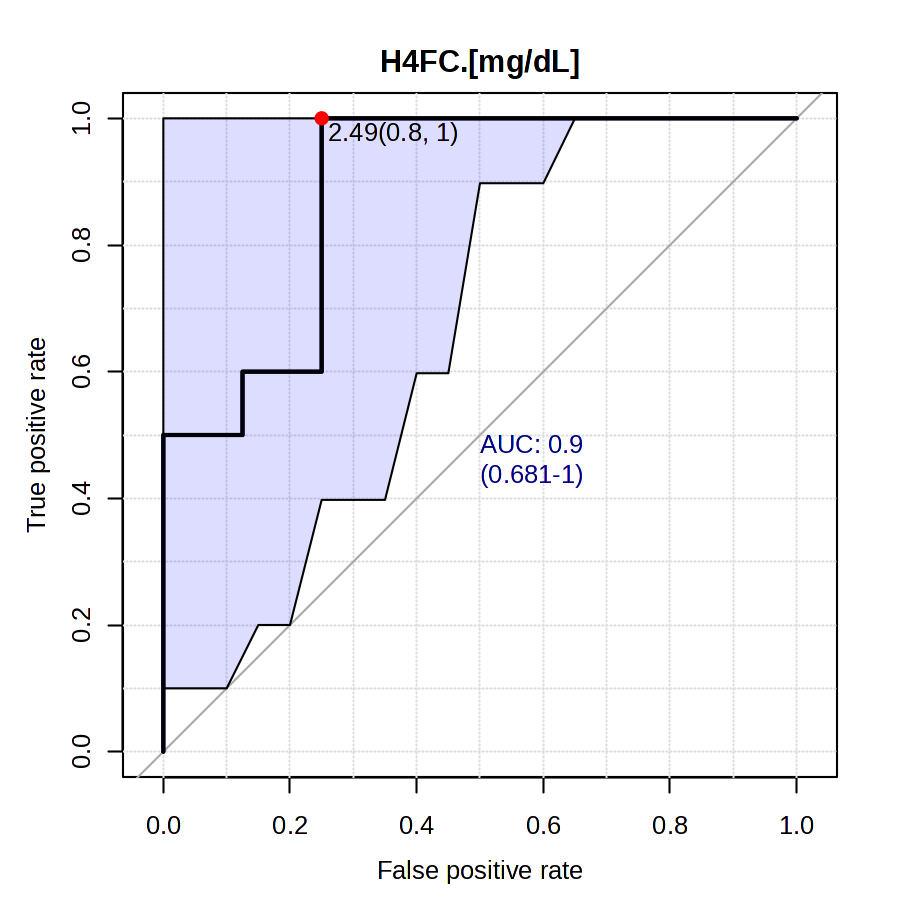


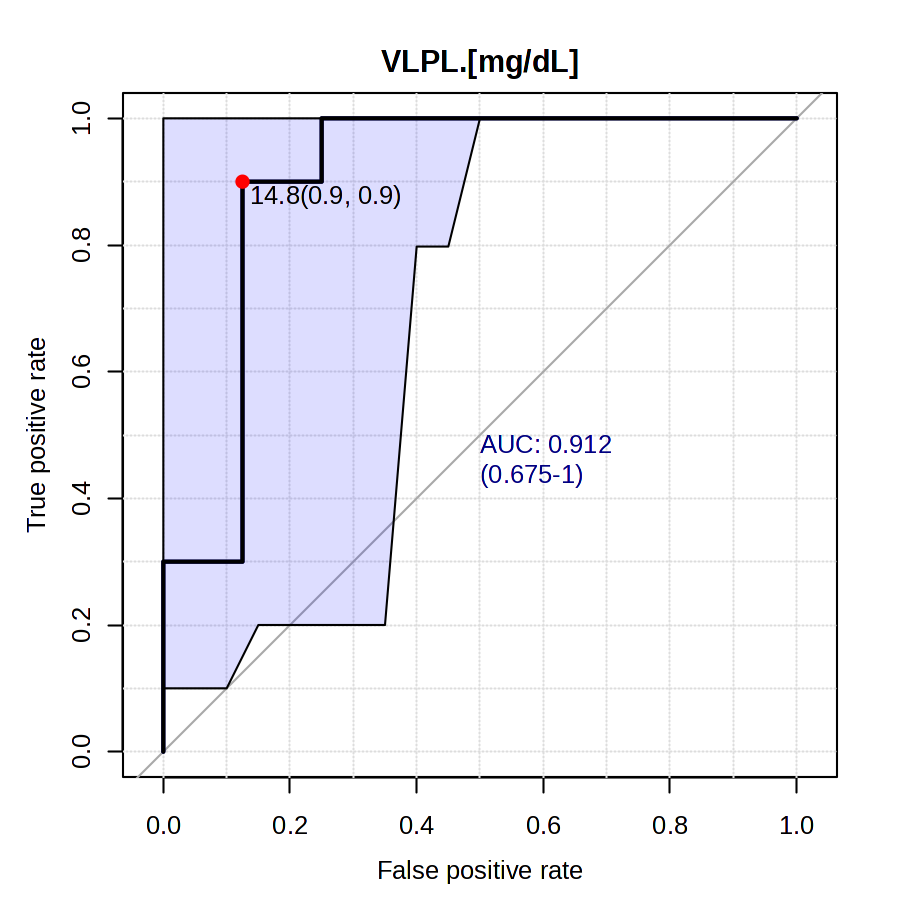


**Supplementary Figure 11: ROC curves for the 3 parameters for females after exclusion of the subjects with antipsychotics**


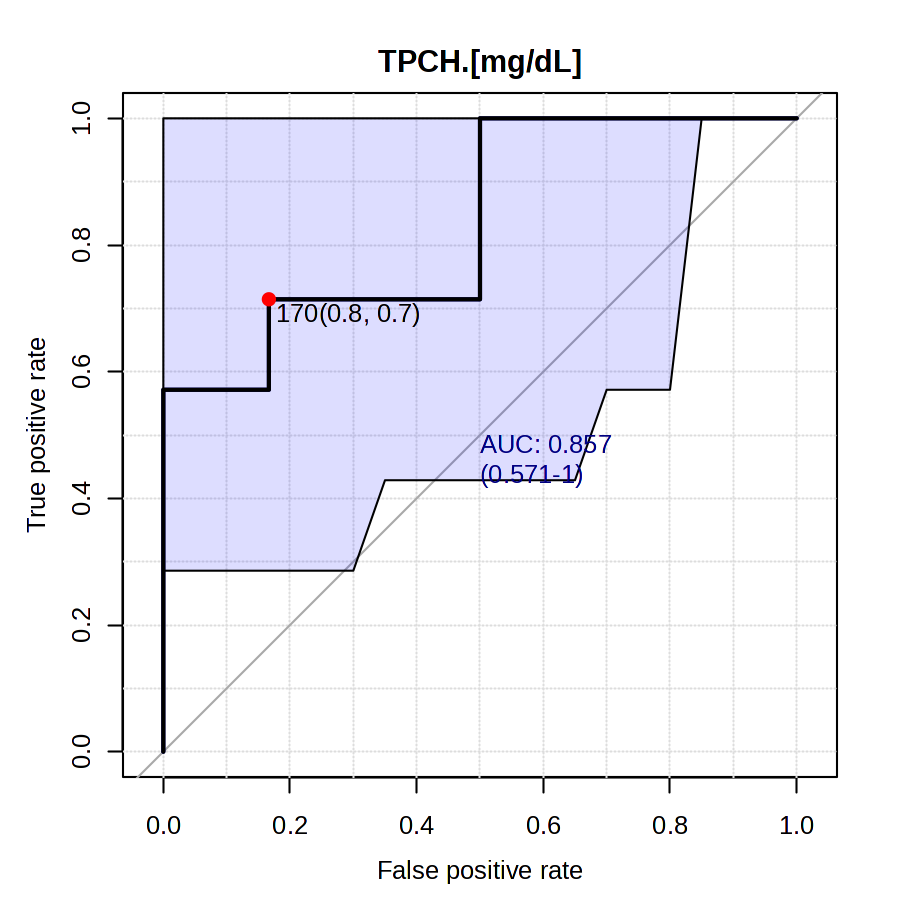


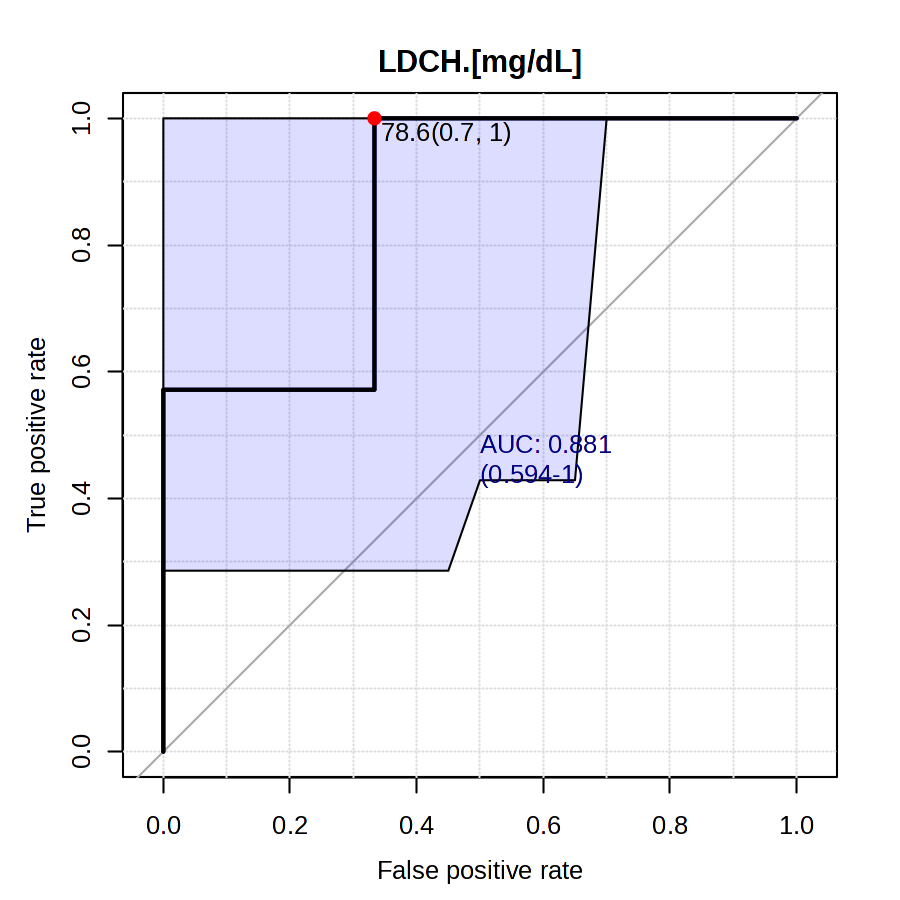


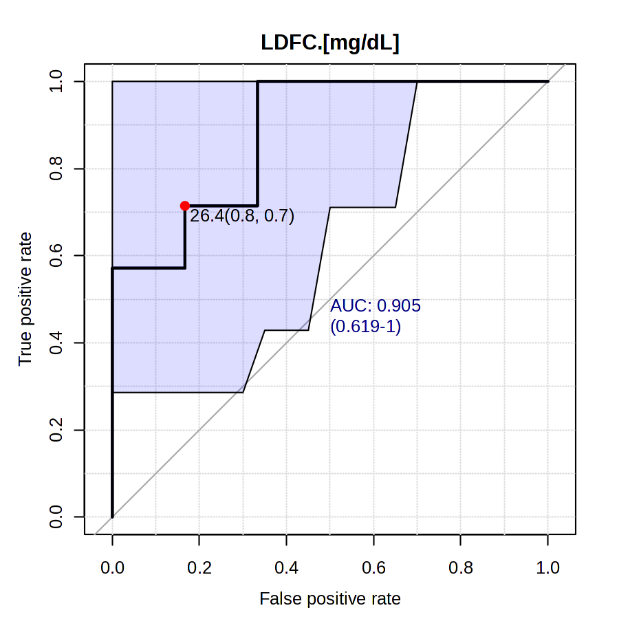


**Supplementary Table 1: Comparison of lipoprotein parameters between UHR-C and UHR-NC**

**1 Moy. : Mean [min-max: Range]**

**2 Wilcoxon rank sum exact test; Wilcoxon rank sum test**

**3 False discovery rate correction for multiple testing**

| **Characteristic** | **UHR-C**, N = 14^1^ | **UHR-NC**, N = 21^1^ | **p-value**^2^ | **q-value**^3^ |
| --- | --- | --- | --- | --- |
| H4A1[mg/dL] | Moy:57 [min-max: 27 - 80] | Moy:74 [min-max: 51 - 95] | **0,0014147** | 0,0646572 |
| H4FC[mg/dL] | Moy:2,41 [min-max: 0,00 - 4,70] | Moy:3,81 [min-max: 2,21 - 6,12] | **0,0023075** | 0,0646572 |
| L4FC[mg/dL] | Moy:1,93 [min-max: 0,00 - 4,24] | Moy:3,89 [min-max: 0,53 - 8,38] | **0,0051876** | 0,0646572 |
| L6CH[mg/dL] | Moy:12,5 [min-max: 1,7 - 23,4] | Moy:18,8 [min-max: 9,6 - 34,4] | **0,0057576** | 0,0646572 |
| TPCH[mg/dL] | Moy:145 [min-max: 87 - 191] | Moy:188 [min-max: 115 - 291] | **0,0069683** | 0,0646572 |
| L6AB[mg/dL] | Moy:10,6 [min-max: 2,0 - 17,9] | Moy:15,7 [min-max: 8,6 - 32,4] | **0,0069683** | 0,0646572 |
| H4CH[mg/dL] | Moy:15,6 [min-max: 6,8 - 24,0] | Moy:20,2 [min-max: 12,3 - 27,9] | **0,0069683** | 0,0646572 |
| L6PN[nmol/L] | Moy:193 [min-max: 36 - 325] | Moy:285 [min-max: 156 - 588] | **0,0069683** | 0,0646572 |
| L5CH[mg/dL] | Moy:7,2 [min-max: 0,0 - 16,1] | Moy:12,4 [min-max: 5,1 - 21,5] | **0,0081939** | 0,0646572 |
| HDFC[mg/dL] | Moy:9,0 [min-max: 4,7 - 18,2] | Moy:12,2 [min-max: 5,0 - 17,7] | **0,0082074** | 0,0646572 |
| L4CH[mg/dL] | Moy:6 [min-max: 0 - 16] | Moy:12 [min-max: 0 - 30] | **0,0085829** | 0,0646572 |
| H4A2[mg/dL] | Moy:16,4 [min-max: 7,7 - 21,8] | Moy:20,5 [min-max: 12,1 - 28,5] | **0,0086248** | 0,0646572 |
| TPAB[mg/dL] | Moy:54 [min-max: 24 - 78] | Moy:75 [min-max: 42 - 134] | **0,0086774** | 0,0646572 |
| TBPN[nmol/L] | Moy:977 [min-max: 427 - 1,413] | Moy:1,368 [min-max: 771 - 2,445] | **0,0086774** | 0,0646572 |
| H3FC[mg/dL] | Moy:1,46 [min-max: 0,42 - 3,35] | Moy:2,16 [min-max: 0,98 - 4,32] | **0,0090465** | 0,0646572 |
| LDFC[mg/dL] | Moy:22 [min-max: 11 - 31] | Moy:30 [min-max: 17 - 53] | **0,0096601** | 0,0646572 |
| L4PL[mg/dL] | Moy:3,6 [min-max: 0,0 - 9,2] | Moy:7,0 [min-max: 0,3 - 15,6] | **0,0099931** | 0,0646572 |
| L5FC[mg/dL] | Moy:2,19 [min-max: 0,00 - 4,12] | Moy:3,60 [min-max: 1,63 - 5,73] | **0,010485** | 0,0646572 |
| H4PL[mg/dL] | Moy:22,6 [min-max: 12,1 - 31,1] | Moy:27,4 [min-max: 17,9 - 38,2] | **0,0119163** | 0,0696164 |
| L5PL[mg/dL] | Moy:4,36 [min-max: 0,00 - 9,01] | Moy:6,94 [min-max: 3,11 - 11,78] | **0,0153208** | 0,0704563 |
| VLFC[mg/dL] | Moy:5,84 [min-max: 2,80 - 11,35] | Moy:8,39 [min-max: 3,98 - 17,47] | **0,0160312** | 0,0704563 |
| TPA1[mg/dL] | Moy:120 [min-max: 87 - 187] | Moy:139 [min-max: 94 - 192] | **0,0161422** | 0,0704563 |
| L4PN[nmol/L] | Moy:71 [min-max: 0 - 181] | Moy:140 [min-max: 11 - 321] | **0,0175241** | 0,0704563 |
| LDCH[mg/dL] | Moy:72 [min-max: 33 - 110] | Moy:101 [min-max: 54 - 180] | **0,0175954** | 0,0704563 |
| TPTG[mg/dL] | Moy:79 [min-max: 43 - 206] | Moy:111 [min-max: 57 - 305] | **0,0178083** | 0,0704563 |
| L4AB[mg/dL] | Moy:3,9 [min-max: 0,0 - 10,0] | Moy:7,7 [min-max: 0,6 - 17,7] | **0,018324** | 0,0704563 |
| IDPL[mg/dL] | Moy:4,19 [min-max: 0,72 - 10,04] | Moy:6,18 [min-max: 1,62 - 17,30] | **0,0184058** | 0,0704563 |
| L6PL[mg/dL] | Moy:7,84 [min-max: 2,66 - 14,12] | Moy:10,69 [min-max: 6,05 - 18,68] | **0,0184139** | 0,0704563 |
| LDPL[mg/dL] | Moy:43 [min-max: 24 - 61] | Moy:57 [min-max: 33 - 94] | **0,0196182** | 0,0704563 |
| L5AB[mg/dL] | Moy:5,6 [min-max: 0,0 - 11,4] | Moy:8,8 [min-max: 4,2 - 15,0] | **0,0210729** | 0,0704563 |
| L5PN[nmol/L] | Moy:101 [min-max: 0 - 208] | Moy:160 [min-max: 75 - 273] | **0,0210729** | 0,0704563 |
| HDA1[mg/dL] | Moy:120 [min-max: 89 - 191] | Moy:140 [min-max: 93 - 194] | **0,0215812** | 0,0704563 |
| LDAB[mg/dL] | Moy:45 [min-max: 20 - 65] | Moy:62 [min-max: 32 - 106] | **0,0215812** | 0,0704563 |
| LDPN[nmol/L] | Moy:811 [min-max: 357 - 1,184] | Moy:1,122 [min-max: 589 - 1,925] | **0,0215812** | 0,0704563 |
| VLTG[mg/dL] | Moy:46 [min-max: 17 - 134] | Moy:71 [min-max: 32 - 203] | **0,0237075** | 0,0751865 |
| IDTG[mg/dL] | Moy:6 [min-max: 0 - 32] | Moy:10 [min-max: 0 - 42] | **0,0324919** | 0,1001834 |
| V5TG[mg/dL] | Moy:1,96 [min-max: 1,29 - 2,39] | Moy:2,34 [min-max: 1,47 - 3,45] | **0,0338699** | 0,1016096 |
| L6FC[mg/dL] | Moy:3,48 [min-max: 0,49 - 7,10] | Moy:4,94 [min-max: 2,73 - 8,60] | **0,0352977** | 0,1031064 |
| V1TG[mg/dL] | Moy:22 [min-max: 3 - 70] | Moy:38 [min-max: 10 - 117] | **0,0383627** | 0,1091863 |
| V2TG[mg/dL] | Moy:7 [min-max: 1 - 27] | Moy:11 [min-max: 4 - 32] | **0,047891** | 0,1328974 |
| L3FC[mg/dL] | Moy:3,44 [min-max: 1,33 - 5,46] | Moy:4,99 [min-max: 2,01 - 10,77] | 0,0508079 | 0,1373917 |
| TPA2[mg/dL] | Moy:29,0 [min-max: 20,1 - 41,3] | Moy:32,9 [min-max: 23,1 - 50,1] | 0,0519861 | 0,1373917 |
| H4TG[mg/dL] | Moy:2,88 [min-max: 1,38 - 5,84] | Moy:3,42 [min-max: 1,82 - 4,78] | 0,0549137 | 0,1408329 |
| L3CH[mg/dL] | Moy:11,5 [min-max: 3,8 - 18,8] | Moy:15,9 [min-max: 6,1 - 38,2] | 0,0563609 | 0,1408329 |
| VLPL[mg/dL] | Moy:14 [min-max: 7 - 28] | Moy:18 [min-max: 8 - 37] | 0,0570944 | 0,1408329 |
| HDA2[mg/dL] | Moy:29,3 [min-max: 20,5 - 41,0] | Moy:33,1 [min-max: 23,5 - 50,7] | 0,0660015 | 0,1592646 |
| H3A1[mg/dL] | Moy:21,1 [min-max: 15,3 - 32,1] | Moy:25,3 [min-max: 15,1 - 43,6] | 0,0712935 | 0,168374 |
| V4TG[mg/dL] | Moy:5,07 [min-max: 1,83 - 9,73] | Moy:6,51 [min-max: 3,61 - 13,52] | 0,089215 | 0,2020993 |
| L3PL[mg/dL] | Moy:6,8 [min-max: 3,0 - 10,3] | Moy:8,9 [min-max: 3,8 - 19,7] | 0,089215 | 0,2020993 |
| V3TG[mg/dL] | Moy:6,6 [min-max: 1,2 - 21,2] | Moy:9,0 [min-max: 2,4 - 27,7] | 0,0959144 | 0,208755 |
| VLPN[nmol/L] | Moy:98 [min-max: 61 - 183] | Moy:125 [min-max: 61 - 244] | 0,0959144 | 0,208755 |
| VLAB[mg/dL] | Moy:5,40 [min-max: 3,38 - 10,09] | Moy:6,89 [min-max: 3,34 - 13,40] | 0,0989365 | 0,2111914 |
| L1FC[mg/dL] | Moy:5,45 [min-max: 3,29 - 7,13] | Moy:6,84 [min-max: 3,01 - 11,41] | 0,1134937 | 0,2376944 |
| H3CH[mg/dL] | Moy:8,44 [min-max: 6,43 - 13,67] | Moy:9,74 [min-max: 5,76 - 17,36] | 0,1183711 | 0,2433184 |
| V1FC[mg/dL] | Moy:1,54 [min-max: 0,33 - 4,12] | Moy:2,51 [min-max: 0,41 - 7,54] | 0,1213795 | 0,244966 |
| L3AB[mg/dL] | Moy:7,0 [min-max: 1,9 - 10,7] | Moy:9,1 [min-max: 3,7 - 20,7] | 0,1266843 | 0,2467011 |
| L3PN[nmol/L] | Moy:126 [min-max: 35 - 194] | Moy:166 [min-max: 67 - 377] | 0,1266843 | 0,2467011 |
| LDHD[-/-] | Moy:1,58 [min-max: 0,71 - 2,68] | Moy:1,96 [min-max: 0,92 - 3,88] | 0,1296607 | 0,2481438 |
| IDAB[mg/dL] | Moy:3,24 [min-max: 1,52 - 5,89] | Moy:4,35 [min-max: 1,74 - 10,34] | 0,1340039 | 0,2505487 |
| IDPN[nmol/L] | Moy:59 [min-max: 28 - 107] | Moy:79 [min-max: 32 - 188] | 0,1354317 | 0,2505487 |
| L1CH[mg/dL] | Moy:19 [min-max: 11 - 25] | Moy:23 [min-max: 10 - 39] | 0,142969 | 0,2548145 |
| L1AB[mg/dL] | Moy:9,8 [min-max: 6,0 - 12,5] | Moy:12,0 [min-max: 5,3 - 20,5] | 0,1446244 | 0,2548145 |

**Supplementary Table 2: ROC curves to predict psychotic transition using lipoprotein parameters at baseline**

|  | AUC | T-test | Log2 FC |
| --- | --- | --- | --- |
| H4A1 [mg/dL] | 0.81293 | 0.000658 | -0.37685 |
| H4FC [mg/dL] | 0.80952 | 0.001051 | -0.65847 |
| L4FC [mg/dL] | 0.78401 | 0.002994 | -1.00230 |
| L6CH [mg/dL] | 0.78061 | 0.003625 | -0.59776 |
| L5FC [mg/dL] | 0.7602 | 0.003705 | -0.71529 |
| H4CH [mg/dL] | 0.76871 | 0.004203 | -0.37014 |
| H4A2 [mg/dL] | 0.76701 | 0.004497 | -0.31483 |
| L5CH [mg/dL] | 0.76871 | 0.004899 | -0.75608 |
| TPCH [mg/dL] | 0.76871 | 0.005365 | -0.37181 |
| L6AB [mg/dL] | 0.76871 | 0.006025 | -0.56485 |
| L6PN [nmol/L] | 0.76871 | 0.006028 | -0.56486 |
| TPAB [mg/dL] | 0.76190 | 0.006759 | -0.48589 |
| TBPN [nmol/L] | 0.76190 | 0.006760 | -0.48589 |
| LDFC [mg/dL] | 0.75850 | 0.007889 | -0.48792 |
| L4CH [mg/dL] | 0.76701 | 0.008055 | -1.13950 |
| L5PL [mg/dL] | 0.74660 | 0.008142 | -0.66778 |
| H4PL [mg/dL] | 0.75170 | 0.008685 | -0.28091 |
| LDCH [mg/dL] | 0.74150 | 0.010606 | -0.49546 |
| L4PL [mg/dL] | 0.76190 | 0.010656 | -0.93805 |
| L5AB [mg/dL] | 0.73469 | 0.010958 | -0.65247 |
| L5PN [nmol/L] | 0.73469 | 0.010970 | -0.65249 |
| L6PL [mg/dL] | 0.73980 | 0.010990 | -0.44726 |
| L6FC [mg/dL] | 0.71429 | 0.011363 | -0.50577 |
| LDPN [nmol/L] | 0.73129 | 0.012228 | -0.46829 |
| LDAB [mg/dL] | 0.73129 | 0.012229 | -0.46828 |
| H3FC [mg/dL] | 0.76531 | 0.012409 | -0.56472 |
| HDFC [mg/dL] | 0.76871 | 0.014205 | -0.44628 |
| L4PN [nmol/L] | 0.74150 | 0.015869 | -0.96219 |
| L4AB [mg/dL] | 0.73980 | 0.015894 | -0.96178 |
| LDPL [mg/dL] | 0.73469 | 0.016080 | -0.39463 |
| Isoleucine [mmol/L] | 0.67857 | 0.024990 | 0.21839 |
| L3FC [mg/dL] | 0.69898 | 0.026898 | -0.53580 |
| VLFC [mg/dL] | 0.74490 | 0.027990 | -0.52432 |
| TPA1 [mg/dL] | 0.74150 | 0.029576 | -0.21725 |
| V5TG [mg/dL] | 0.71599 | 0.030538 | -0.25524 |
| HDA1 [mg/dL] | 0.73129 | 0.038848 | -0.21505 |
| H3A1 [mg/dL] | 0.68367 | 0.046589 | -0.26454 |

**Supplementary Table 3:** **Comparison of lipoprotein parameters between Women and Men**

**1 Moy (min, max)**

**2 Wilcoxon rank sum exact test; Wilcoxon rank sum test**

**3 False discovery rate correction for multiple testing**

| Characteristic | F, N = 14^1^ | M, N = 21^1^ | p-value^2^ | q-value^3^ |
| --- | --- | --- | --- | --- |
| HDA1.[mg/dL] | 144 (128, 160) | 119 (103, 141) | 0.003 | 0.14 |
| HDPL.[mg/dL] | 77 (68, 85) | 63 (58, 72) | 0.005 | 0.14 |
| L3TG.[mg/dL] | 2.11 (1.76, 2.42) | 1.61 (1.35, 1.84) | 0.005 | 0.14 |
| L3FC.[mg/dL] | 4.53 (4.08, 6.40) | 3.24 (2.49, 4.52) | 0.005 | 0.14 |
| HDCH.[mg/dL] | 55 (51, 67) | 45 (36, 55) | 0.007 | 0.14 |
| TPA1.[mg/dL] | 141 (126, 158) | 119 (104, 141) | 0.007 | 0.14 |
| L2FC.[mg/dL] | 6.07 (5.01, 6.85) | 4.37 (3.70, 5.27) | 0.007 | 0.14 |
| H3FC.[mg/dL] | 2.16 (1.73, 2.80) | 1.62 (1.13, 2.05) | 0.008 | 0.15 |
| V1CH.[mg/dL] | 3.7 (2.5, 5.9) | 6.5 (4.6, 9.4) | 0.012 | 0.2 |
| HDFC.[mg/dL] | 12.6 (10.6, 15.9) | 9.4 (6.5, 11.6) | 0.014 | 0.2 |
| H2A1.[mg/dL] | 17.3 (15.6, 20.7) | 13.9 (11.9, 18.1) | 0.015 | 0.2 |
| Lysine.(rawConc).[mmol/L] | 0.20 (0.18, 0.24) | 0.18 (0.00, 0.19) | 0.016 | 0.2 |
| L2TG.[mg/dL] | 2.18 (1.66, 2.58) | 1.50 (1.36, 1.73) | 0.017 | 0.2 |
| L1PL.[mg/dL] | 13.3 (11.6, 14.7) | 11.1 (10.2, 12.3) | 0.018 | 0.2 |
| L1AB.[mg/dL] | 12.4 (10.4, 13.7) | 9.8 (8.6, 10.9) | 0.019 | 0.2 |
| L1PN.[nmol/L] | 225 (189, 248) | 178 (156, 198) | 0.019 | 0.2 |
| L2PL.[mg/dL] | 11.03 (8.17, 12.75) | 8.90 (6.61, 9.64) | 0.020 | 0.2 |
| H4FC.[mg/dL] | 4.24 (2.95, 4.61) | 2.84 (2.48, 3.81) | 0.022 | 0.2 |
| TPCH.[mg/dL] | 170 (154, 215) | 140 (129, 183) | 0.025 | 0.2 |
| LDFC.[mg/dL] | 26 (24, 35) | 23 (18, 28) | 0.025 | 0.2 |
| L2AB.[mg/dL] | 10.8 (8.0, 12.5) | 8.3 (6.6, 9.6) | 0.025 | 0.2 |
| H1CH.[mg/dL] | 17 (15, 20) | 13 (7, 17) | 0.025 | 0.2 |
| L2PN.[nmol/L] | 196 (145, 227) | 152 (119, 174) | 0.025 | 0.2 |
| LDPL.[mg/dL] | 50 (46, 64) | 45 (36, 54) | 0.027 | 0.2 |
| V1PL.[mg/dL] | 3.1 (2.4, 6.1) | 4.6 (3.4, 8.8) | 0.029 | 0.2 |
| L2CH.[mg/dL] | 20.2 (14.8, 23.4) | 15.8 (12.1, 16.9) | 0.029 | 0.2 |
| L3PL.[mg/dL] | 7.99 (6.98, 10.64) | 6.64 (4.99, 8.24) | 0.029 | 0.2 |
| Asparagine.(rawConc).[mmol/L] |  |  | 0.030 | 0.2 |
| LDCH.[mg/dL] | 86 (79, 116) | 77 (61, 95) | 0.031 | 0.2 |
| H1A1.[mg/dL] | 29 (22, 33) | 19 (12, 30) | 0.032 | 0.2 |
| L3CH.[mg/dL] | 13.6 (11.9, 19.6) | 11.6 (8.3, 15.0) | 0.040 | 0.2 |
| H2CH.[mg/dL] | 8.02 (6.99, 9.49) | 6.53 (4.61, 7.96) | 0.040 | 0.2 |
| H3PL.[mg/dL] | 15.96 (13.63, 17.01) | 12.60 (10.73, 15.75) | 0.040 | 0.2 |
| Weight | 60 (54, 69) | 70 (61, 74) | 0.043 | 0.2 |
| LDAB.[mg/dL] | 54 (48, 70) | 47 (36, 60) | 0.044 | 0.2 |
| L3AB.[mg/dL] | 8.3 (6.9, 10.8) | 7.0 (5.2, 8.5) | 0.044 | 0.2 |
| H3A1.[mg/dL] | 25.9 (21.0, 29.2) | 20.5 (16.4, 25.2) | 0.044 | 0.2 |
| LDPN.[nmol/L] | 974 (878, 1,280) | 861 (650, 1,092) | 0.044 | 0.2 |
| L3PN.[nmol/L] | 150 (126, 197) | 127 (94, 154) | 0.044 | 0.2 |
| L1CH.[mg/dL] | 23 (20, 27) | 20 (17, 23) | 0.045 | 0.2 |
| L1FC.[mg/dL] | 6.79 (5.83, 8.05) | 5.41 (4.60, 6.41) | 0.045 | 0.2 |
| H1TG.[mg/dL] | 2.94 (2.33, 4.06) | 2.46 (1.74, 2.96) | 0.047 | 0.2 |
| Creatine.(rawConc).[mmol/L] | 0.020 (0.012, 0.022) | 0.012 (0.008, 0.016) | 0.048 | 0.2 |
| H1PL.[mg/dL] | 22 (18, 25) | 15 (10, 23) | 0.051 | 0.2 |
| V1FC.[mg/dL] | 0.96 (0.62, 2.35) | 1.74 (1.09, 3.76) | 0.052 | 0.2 |
| H1FC.[mg/dL] | 4.53 (3.79, 5.44) | 3.61 (2.32, 4.86) | 0.052 | 0.2 |
| H2PL.[mg/dL] | 12.1 (10.8, 14.6) | 10.8 (7.6, 13.0) | 0.057 | 0.2 |
| H4CH.[mg/dL] | 21.1 (15.2, 23.3) | 17.7 (16.4, 20.3) | 0.059 | 0.2 |
| TPAB.[mg/dL] | 65 (59, 81) | 59 (45, 69) | 0.063 | 0.2 |
| H3CH.[mg/dL] | 9.41 (8.13, 11.75) | 8.82 (7.07, 10.22) | 0.063 | 0.2 |
| TBPN.[nmol/L] | 1,185 (1,075, 1,468) | 1,066 (815, 1,263) | 0.063 | 0.2 |
| L1TG.[mg/dL] | 4.79 (3.64, 5.26) | 3.17 (2.90, 4.52) | 0.064 | 0.2 |
| H4A1.[mg/dL] | 76 (67, 80) | 67 (58, 73) | 0.068 | 0.2 |
| Creatinine.(rawConc).[mmol/L] | 0.087 (0.076, 0.098) | 0.070 (0.063, 0.085) | 0.075 | 0.2 |
| HDA2.[mg/dL] | 33.4 (30.0, 35.9) | 30.2 (26.8, 31.9) | 0.078 | 0.2 |
| V3FC.[mg/dL] | 0.84 (0.50, 1.28) | 1.15 (0.86, 2.07) | 0.078 | 0.2 |
| L4FC.[mg/dL] | 3.17 (2.55, 5.23) | 2.14 (1.44, 3.60) | 0.078 | 0.2 |
| LDTG.[mg/dL] | 15.0 (11.6, 16.5) | 12.2 (11.0, 15.3) | 0.081 | 0.2 |
| VLCH.[mg/dL] | 14 (10, 17) | 17 (14, 25) | 0.083 | 0.2 |
| TPA2.[mg/dL] | 33.5 (29.8, 35.6) | 29.9 (26.7, 32.0) | 0.089 | 0.2 |
| H4PL.[mg/dL] | 28.0 (24.3, 29.8) | 25.4 (22.9, 27.9) | 0.089 | 0.2 |
| V2FC.[mg/dL] | 0.72 (0.45, 1.08) | 1.01 (0.76, 1.70) | 0.092 | 0.2 |
| Methionine.(rawConc).[mmol/L] | 0.049 (0.030, 0.057) | 0.032 (0.015, 0.050) | 0.092 | 0.2 |
| L5FC.[mg/dL] | 3.43 (2.63, 4.58) | 2.86 (2.01, 3.49) | 0.095 | 0.2 |
| Trimethylamine-N-oxide.(rawConc).[mmol/L] | 0.016 (0.006, 0.019) | 0.020 (0.013, 0.033) | 0.10 | 0.2 |
| Threonine.(rawConc).[mmol/L] | 0.16 (0.16, 0.18) | 0.11 (0.00, 0.18) | 0.12 | 0.3 |
| Acetic.acid.(rawConc).[mmol/L] | 0.022 (0.016, 0.033) | 0.033 (0.023, 0.055) | 0.12 | 0.3 |
| V3CH.[mg/dL] | 2.42 (1.39, 3.18) | 2.77 (2.01, 4.29) | 0.13 | 0.3 |
| H1A2.[mg/dL] | 2.39 (2.20, 3.31) | 2.17 (1.37, 2.96) | 0.13 | 0.3 |
| L4PL.[mg/dL] | 6.5 (3.6, 8.9) | 4.0 (2.4, 7.0) | 0.14 | 0.3 |
| H4A2.[mg/dL] | 20.1 (18.4, 21.9) | 19.1 (16.6, 20.7) | 0.14 | 0.3 |
| L5PL.[mg/dL] | 6.22 (4.86, 8.83) | 5.28 (3.61, 6.88) | 0.15 | 0.3 |
| L4AB.[mg/dL] | 7.1 (3.8, 9.6) | 4.5 (2.3, 7.7) | 0.2 | 0.5 |
| PANSS_NEGATIVE | 11.0 (8.0, 17.3) | 14.0 (11.0, 21.0) | 0.2 | 0.4 |
| V1TG.[mg/dL] | 20 (10, 35) | 21 (19, 43) | 0.2 | 0.4 |
| V5TG.[mg/dL] | 2.24 (1.84, 2.70) | 2.08 (1.74, 2.39) | 0.2 | 0.4 |
| V2CH.[mg/dL] | 2.02 (1.41, 2.75) | 2.36 (1.85, 3.24) | 0.2 | 0.4 |
| L4CH.[mg/dL] | 11 (5, 15) | 6 (3, 12) | 0.2 | 0.4 |
| L5CH.[mg/dL] | 11.3 (7.7, 16.0) | 9.3 (5.6, 12.0) | 0.2 | 0.4 |
| L6FC.[mg/dL] | 4.75 (3.58, 5.87) | 3.95 (3.10, 4.75) | 0.2 | 0.4 |
| L5AB.[mg/dL] | 7.6 (6.0, 11.2) | 7.2 (4.3, 8.5) | 0.2 | 0.4 |
| H2FC.[mg/dL] | 1.98 (1.65, 2.42) | 1.85 (1.26, 2.13) | 0.2 | 0.4 |
| H2A2.[mg/dL] | 3.23 (2.72, 3.88) | 2.93 (2.17, 3.68) | 0.2 | 0.4 |
| H3A2.[mg/dL] | 6.09 (5.12, 7.28) | 5.36 (4.80, 6.69) | 0.2 | 0.4 |
| L4PN.[nmol/L] | 130 (68, 175) | 82 (41, 139) | 0.2 | 0.4 |
| L5PN.[nmol/L] | 138 (108, 203) | 131 (79, 154) | 0.2 | 0.4 |
| VLPL.[mg/dL] | 13 (11, 19) | 15 (12, 23) | 0.3 | 0.6 |
| L6TG.[mg/dL] | 2.85 (2.71, 3.47) | 2.71 (1.78, 3.33) | 0.3 | 0.6 |
| Choline.(rawConc).[mmol/L] |  |  | 0.3 | 0.6 |
| Ca-EDTA.(rawConc).[mmol/L] |  |  | 0.3 | 0.6 |
| HDTG.[mg/dL] | 9.52 (8.32, 10.42) | 8.23 (7.14, 9.80) | 0.3 | 0.5 |
| L4TG.[mg/dL] | 1.56 (1.00, 2.00) | 1.47 (0.82, 1.73) | 0.3 | 0.5 |
| V4TG.[mg/dL] | 6.16 (4.94, 7.11) | 6.01 (3.75, 7.08) | 0.4 | 0.7 |
| V5FC.[mg/dL] | 0.52 (0.38, 0.62) | 0.61 (0.37, 0.79) | 0.4 | 0.7 |
| L6CH.[mg/dL] | 17.2 (14.2, 20.8) | 14.2 (12.5, 19.3) | 0.4 | 0.7 |
| Tyrosine.(rawConc).[mmol/L] | 0.052 (0.046, 0.062) | 0.058 (0.052, 0.062) | 0.4 | 0.7 |
| PANSS_GENERAL | 35 (31, 40) | 40 (31, 46) | 0.4 | 0.6 |
| PANSS_TOTAL | 60 (52, 70) | 64 (53, 82) | 0.4 | 0.6 |
| V4FC.[mg/dL] | 1.28 (0.95, 1.96) | 1.55 (1.25, 2.23) | 0.4 | 0.6 |
| L6PL.[mg/dL] | 9.59 (8.31, 11.74) | 8.42 (7.28, 10.83) | 0.4 | 0.6 |
| BMI | 23.2 (20.6, 24.8) | 21.3 (20.4, 22.8) | 0.5 | 0.7 |
| VLFC.[mg/dL] | 6.23 (4.83, 8.03) | 6.61 (5.04, 9.60) | 0.5 | 0.7 |
| VLAB.[mg/dL] | 5.43 (4.10, 6.54) | 5.43 (4.62, 8.07) | 0.5 | 0.7 |
| V5CH.[mg/dL] | 1.17 (0.98, 1.51) | 1.12 (0.78, 1.33) | 0.5 | 0.7 |
| V2PL.[mg/dL] | 2.17 (1.44, 3.11) | 2.10 (1.57, 3.82) | 0.5 | 0.7 |
| V3PL.[mg/dL] | 2.55 (1.68, 3.30) | 2.43 (1.94, 4.17) | 0.5 | 0.7 |
| L6AB.[mg/dL] | 13.5 (11.8, 16.0) | 11.3 (10.4, 16.4) | 0.5 | 0.7 |
| H2TG.[mg/dL] | 1.46 (1.27, 1.84) | 1.39 (1.25, 1.72) | 0.5 | 0.7 |
| VLPN.[nmol/L] | 99 (75, 119) | 99 (84, 147) | 0.5 | 0.7 |
| L6PN.[nmol/L] | 245 (215, 290) | 205 (188, 298) | 0.5 | 0.7 |
| Glycine.(rawConc).[mmol/L] | 0.30 (0.29, 0.33) | 0.28 (0.25, 0.34) | 0.5 | 0.7 |
| Ornithine.(rawConc).[mmol/L] | 0.026 (0.010, 0.047) | 0.025 (0.013, 0.050) | 0.5 | 0.7 |
| Valine.(rawConc).[mmol/L] | 0.250 (0.226, 0.268) | 0.241 (0.227, 0.253) | 0.5 | 0.7 |
| Glucose.(rawConc).[mmol/L] | 4.62 (4.27, 4.90) | 4.70 (4.46, 4.95) | 0.5 | 0.7 |
| K-EDTA.(rawConc).[mmol/L] | 0.02 (0.02, 0.02) | 0.02 (0.02, 0.02) | 0.5 | 0.7 |
| Age | 19.5 (18.0, 22.5) | 20.0 (19.0, 23.0) | 0.6 | 0.8 |
| IDPL.[mg/dL] | 4.82 (3.74, 6.09) | 3.90 (3.41, 5.97) | 0.6 | 0.8 |
| V5PL.[mg/dL] | 1.39 (1.15, 1.71) | 1.28 (1.10, 1.60) | 0.6 | 0.8 |
| 2-Aminobutyric.acid.(rawConc).[mmol/L] | 0.027 (0.000, 0.034) | 0.023 (0.000, 0.034) | 0.6 | 0.8 |
| Histidine.(rawConc).[mmol/L] | 0.087 (0.078, 0.091) | 0.084 (0.077, 0.096) | 0.6 | 0.8 |
| 2-Hydroxybutyric.acid.(rawConc).[mmol/L] | 0.000 (0.000, 0.003) | 0.000 (0.000, 0.010) | 0.6 | 0.8 |
| Succinic.acid.(rawConc).[mmol/L] | 0.003 (0.003, 0.003) | 0.003 (0.002, 0.006) | 0.6 | 0.8 |
| Dimethylsulfone.(rawConc).[mmol/L] | 0.007 (0.006, 0.009) | 0.008 (0.006, 0.010) | 0.6 | 0.8 |
| VLTG.[mg/dL] | 48 (36, 67) | 48 (37, 81) | 0.7 | 0.9 |
| ABA1.[-/-] | 0.51 (0.42, 0.59) | 0.48 (0.39, 0.57) | 0.7 | 0.9 |
| Ethanol.(rawConc).[mmol/L] |  |  | 0.7 | 0.9 |
| Acetone.(rawConc).[mmol/L] | 0.026 (0.017, 0.056) | 0.021 (0.017, 0.033) | 0.7 | 0.9 |
| IDAB.[mg/dL] | 3.40 (2.57, 4.56) | 3.45 (2.53, 4.39) | 0.7 | 0.8 |
| L5TG.[mg/dL] | 1.35 (1.19, 1.85) | 1.62 (0.93, 1.86) | 0.7 | 0.8 |
| IDPN.[nmol/L] | 62 (47, 83) | 63 (46, 80) | 0.7 | 0.8 |
| Glutamic.acid.(rawConc).[mmol/L] | 0.09 (0.04, 0.12) | 0.07 (0.04, 0.13) | 0.7 | 0.8 |
| Proline.(rawConc).[mmol/L] | 0.00 (0.00, 0.21) | 0.00 (0.00, 0.21) | 0.7 | 0.8 |
| 3-Hydroxybutyric.acid.(rawConc).[mmol/L] | 0.03 (0.03, 0.06) | 0.03 (0.02, 0.07) | 0.7 | 0.8 |
| TPTG.[mg/dL] | 79 (61, 98) | 80 (62, 118) | 0.8 | >0.9 |
| V4CH.[mg/dL] | 3.99 (2.68, 4.79) | 3.64 (3.08, 5.12) | 0.8 | >0.9 |
| H4TG.[mg/dL] | 3.13 (2.62, 3.76) | 3.29 (2.64, 3.75) | 0.8 | >0.9 |
| LDHD.[-/-] | 1.62 (1.48, 2.07) | 1.76 (1.20, 2.09) | 0.8 | >0.9 |
| Alanine.(rawConc).[mmol/L] | 0.42 (0.37, 0.46) | 0.42 (0.34, 0.45) | 0.8 | >0.9 |
| N,N-Dimethylglycine.(rawConc).[mmol/L] | 0.0060 (0.0050, 0.0070) | 0.0060 (0.0040, 0.0070) | 0.8 | >0.9 |
| Acetoacetic.acid.(rawConc).[mmol/L] | 0.006 (0.005, 0.011) | 0.008 (0.004, 0.013) | 0.8 | >0.9 |
| Pyruvic.acid.(rawConc).[mmol/L] | 0.06 (0.03, 0.09) | 0.05 (0.04, 0.08) | 0.8 | >0.9 |
| Glycerol.(rawConc).[mmol/L] |  |  | 0.8 | >0.9 |
| IDTG.[mg/dL] | 6 (2, 8) | 4 (2, 11) | 0.9 | >0.9 |
| IDCH.[mg/dL] | 9.0 (5.8, 12.5) | 7.9 (6.4, 13.8) | 0.9 | >0.9 |
| IDFC.[mg/dL] | 2.49 (1.64, 3.46) | 2.52 (1.89, 3.98) | 0.9 | >0.9 |
| Glutamine.(rawConc).[mmol/L] | 0.71 (0.67, 0.79) | 0.72 (0.67, 0.78) | 0.9 | >0.9 |
| Phenylalanine.(rawConc).[mmol/L] | 0.063 (0.048, 0.071) | 0.053 (0.044, 0.069) | 0.9 | >0.9 |
| Formic.acid.(rawConc).[mmol/L] | 0.019 (0.016, 0.023) | 0.020 (0.015, 0.021) | 0.9 | >0.9 |
| Lactic.acid.(rawConc).[mmol/L] | 2.17 (1.99, 2.43) | 2.08 (1.85, 2.58) | 0.9 | >0.9 |
| V2TG.[mg/dL] | 8 (5, 11) | 7 (4, 14) | >0.9 | >0.9 |
| V3TG.[mg/dL] | 7.0 (4.4, 9.5) | 6.1 (4.0, 11.2) | >0.9 | >0.9 |
| V4PL.[mg/dL] | 3.23 (2.77, 4.13) | 3.55 (2.57, 4.26) | >0.9 | >0.9 |
| H3TG.[mg/dL] | 1.78 (1.46, 2.02) | 1.84 (1.42, 2.15) | >0.9 | >0.9 |
| Isoleucine.(rawConc).[mmol/L] | 0.064 (0.051, 0.074) | 0.064 (0.058, 0.069) | >0.9 | >0.9 |
| Leucine.(rawConc).[mmol/L] | 0.119 (0.101, 0.128) | 0.116 (0.101, 0.133) | >0.9 | >0.9 |
| Sarcosine.(rawConc).[mmol/L] | 0.004 (0.003, 0.008) | 0.004 (0.003, 0.007) | >0.9 | >0.9 |
| Citric.acid.(rawConc).[mmol/L] | 0.124 (0.108, 0.154) | 0.135 (0.111, 0.141) | >0.9 | >0.9 |

**Supplementary Table 4: Comparison of lipoprotein parameters between UHR-C and UHR-NC after exclusion of subjects with antipsychotics**

**1 Moy (min, max)**

**2 Wilcoxon rank sum exact test; Wilcoxon rank sum test**

**3 False discovery rate correction for multiple testing**

| **Characteristic** | **UHR-C, N = 14^1^** | **UHR-NC, N = 17^1^** | **p-value^2^** | **q-value3** |
| --- | --- | --- | --- | --- |
| PANSS_POSITIVE | 17.5 (15.5, 21.8) | 7.0 (7.0, 13.0) | **<0.001** | 0.004 |
| PANSS_NEGATIVE | 18.5 (13.0, 22.0) | 11.0 (9.0, 13.0) | **0.025** | 0.2 |
| PANSS_GENERAL | 42 (39, 46) | 32 (28, 38) | **0.002** | 0.12 |
| PANSS_TOTAL | 82 (66, 89) | 53 (45, 63) | **<0.001** | 0.014 |
| Antipsychotics | 0 (0%) | 0 (0%) |  |  |
| Age | 20.5 (19.0, 22.5) | 20.0 (18.0, 23.0) | >0.9 | >0.9 |
| Sex |  |  | >0.9 | >0.9 |
| F | 6 (43%) | 7 (41%) |  |  |
| M | 8 (57%) | 10 (59%) |  |  |
| Size | 172 (167, 175) | 174 (170, 181) | 0.3 | 0.5 |
| Weight | 66 (54, 72) | 72 (60, 73) | 0.3 | 0.5 |
| BMI | 23.1 (18.6, 25.5) | 21.1 (20.4, 23.8) | >0.9 | >0.9 |
| VLFC.[mg/dL] | 5.30 (4.23, 6.31) | 7.85 (6.61, 10.38) | **0,008** | 0.2 |
| H4FC.[mg/dL] | 2.41 (2.10, 3.17) | 3.74 (2.72, 4.21) | **0,009** | 0.2 |
| H4A1.[mg/dL] | 58 (50, 69) | 70 (65, 78) | **0,010** | 0.2 |
| L4CH.[mg/dL] | 3.3 (0.7, 9.8) | 11.0 (6.2, 14.2) | **0,015** | 0.2 |
| L4FC.[mg/dL] | 1.72 (0.99, 3.17) | 3.12 (2.37, 4.96) | **0,015** | 0.2 |
| L4PL.[mg/dL] | 2.7 (1.3, 5.8) | 6.5 (4.0, 7.8) | **0,018** | 0.2 |
| VLPL.[mg/dL] | 12.2 (10.7, 14.1) | 17.8 (14.9, 22.5) | **0,025** | 0.2 |
| TPTG.[mg/dL] | 66 (56, 79) | 95 (75, 118) | **0,026** | 0.2 |
| VLTG.[mg/dL] | 39 (28, 49) | 63 (42, 81) | **0,026** | 0.2 |
| HDFC.[mg/dL] | 8.1 (6.7, 10.4) | 11.5 (9.4, 13.2) | **0,030** | 0.2 |
| IDPL.[mg/dL] | 3.44 (2.71, 4.99) | 5.71 (4.01, 6.43) | **0,030** | 0.2 |
| L5CH.[mg/dL] | 6.9 (4.2, 10.2) | 11.2 (8.7, 14.1) | **0,030** | 0.2 |
| L4PN.[nmol/L] | 52 (6, 119) | 130 (82, 164) | **0,030** | 0.2 |
| V2TG.[mg/dL] | 4.8 (2.3, 9.7) | 8.4 (6.7, 14.2) | **0,032** | 0.2 |
| V3TG.[mg/dL] | 4.8 (3.6, 8.2) | 8.4 (6.1, 11.2) | **0,032** | 0.2 |
| L6CH.[mg/dL] | 12.4 (8.3, 16.7) | 17.2 (13.3, 19.3) | **0,032** | 0.2 |
| L4AB.[mg/dL] | 2.9 (0.3, 6.5) | 7.1 (4.5, 9.0) | **0,032** | 0.2 |
| L5FC.[mg/dL] | 2.45 (1.26, 3.36) | 3.21 (2.74, 4.09) | **0,035** | 0.2 |
| H4CH.[mg/dL] | 15.8 (13.8, 19.2) | 18.7 (17.5, 22.0) | **0,036** | 0.2 |
| H3FC.[mg/dL] | 1.39 (1.02, 1.73) | 1.96 (1.43, 2.35) | **0,037** | 0.2 |
| H4A2.[mg/dL] | 17.4 (14.5, 19.7) | 19.7 (17.9, 21.5) | **0,039** | 0.2 |
| TPCH.[mg/dL] | 141 (125, 168) | 163 (140, 206) | **0,040** | 0.2 |
| TPAB.[mg/dL] | 57 (43, 64) | 65 (58, 78) | **0,040** | 0.2 |
| TBPN.[nmol/L] | 1,029 (780, 1,171) | 1,183 (1,047, 1,414) | **0,040** | 0.2 |
| VLPN.[nmol/L] | 85 (74, 112) | 123 (95, 150) | **0,040** | 0.2 |
| VLAB.[mg/dL] | 4.67 (4.08, 6.14) | 6.74 (5.22, 8.26) | **0,043** | 0.2 |
| V5TG.[mg/dL] | 2.07 (1.77, 2.19) | 2.42 (1.74, 2.54) | **0,043** | 0.2 |
| L6AB.[mg/dL] | 10.5 (7.8, 13.5) | 13.7 (11.3, 15.5) | **0,044** | 0.2 |
| L6PN.[nmol/L] | 191 (142, 246) | 249 (205, 281) | **0,044** | 0.2 |
| LDFC.[mg/dL] | 23 (18, 25) | 26 (22, 33) | **0,048** | 0.2 |
| L5PL.[mg/dL] | 4.51 (2.74, 5.89) | 6.19 (5.09, 7.70) | 0,052 | 0.2 |
| H4TG.[mg/dL] | 2.63 (1.91, 3.23) | 3.62 (2.75, 3.93) | 0,052 | 0.2 |
| H4PL.[mg/dL] | 23.0 (20.8, 26.7) | 27.0 (23.4, 28.5) | 0,053 | 0.2 |
| IDTG.[mg/dL] | 3 (1, 7) | 8 (4, 11) | 0,054 | 0.2 |
| V1TG.[mg/dL] | 20 (10, 22) | 35 (19, 45) | 0,064 | 0.3 |
| V4TG.[mg/dL] | 4.94 (3.82, 6.56) | 6.51 (5.43, 7.12) | 0,064 | 0.3 |
| LDCH.[mg/dL] | 73 (62, 85) | 84 (77, 109) | 0,071 | 0.3 |
| TPA1.[mg/dL] | 118 (101, 131) | 135 (119, 151) | 0,071 | 0.3 |
| L5AB.[mg/dL] | 5.7 (3.4, 7.8) | 7.6 (6.6, 9.8) | 0,071 | 0.3 |
| L5PN.[nmol/L] | 104 (61, 141) | 138 (120, 177) | 0,071 | 0.3 |
| 2-Hydroxybutyric.acid.(rawConc).[mmol/L] | 0.000 (0.000, 0.000) | 0.000 (0.000, 0.016) | 0,078 | 0.3 |
| L6PL.[mg/dL] | 7.83 (5.67, 9.55) | 9.70 (7.48, 10.83) | 0,081 | 0.3 |
| HDA1.[mg/dL] | 115 (101, 134) | 138 (119, 152) | 0,084 | 0.3 |
| LDPL.[mg/dL] | 44 (37, 51) | 50 (45, 61) | 0,092 | 0.3 |
| V2CH.[mg/dL] | 2.10 (1.61, 2.54) | 3.07 (2.05, 3.72) | 0,092 | 0.3 |
| LDAB.[mg/dL] | 47 (35, 55) | 52 (47, 66) | 0,100 | 0.3 |
| V1FC.[mg/dL] | 1.13 (0.83, 1.74) | 2.16 (1.50, 3.50) | 0,100 | 0.3 |
| V2FC.[mg/dL] | 0.82 (0.58, 0.99) | 1.24 (0.79, 1.61) | 0,100 | 0.3 |
| LDPN.[nmol/L] | 864 (645, 1,005) | 944 (856, 1,205) | 0,100 | 0.3 |
| V2PL.[mg/dL] | 1.86 (1.46, 2.78) | 2.62 (2.02, 3.82) | 0,110 | 0.3 |
| Leucine.(rawConc).[mmol/L] | 0.122 (0.116, 0.132) | 0.108 (0.091, 0.133) | 0,110 | 0.3 |
| LDHD.[-/-] | 1.58 (1.18, 1.93) | 1.81 (1.56, 2.31) | 0,120 | 0.3 |
| Creatinine.(rawConc).[mmol/L] | 0.085 (0.076, 0.099) | 0.076 (0.061, 0.088) | 0,120 | 0.3 |
| V4FC.[mg/dL] | 1.37 (0.92, 1.64) | 1.63 (1.25, 2.23) | 0,130 | 0.4 |
| V1CH.[mg/dL] | 5.7 (3.7, 6.4) | 7.0 (4.6, 8.8) | 0,140 | 0.4 |
| V3PL.[mg/dL] | 2.22 (1.98, 3.11) | 3.28 (2.43, 4.17) | 0,140 | 0.4 |
| L6FC.[mg/dL] | 3.58 (2.50, 4.56) | 4.44 (3.57, 4.84) | 0,140 | 0.4 |
| Isoleucine.(rawConc).[mmol/L] | 0.066 (0.060, 0.080) | 0.062 (0.051, 0.069) | 0,140 | 0.4 |
| VLCH.[mg/dL] | 14 (11, 18) | 17 (15, 25) | 0,150 | 0.4 |
| Phenylalanine.(rawConc).[mmol/L] | 0.067 (0.054, 0.077) | 0.064 (0.042, 0.069) | 0,150 | 0.4 |
| TPA2.[mg/dL] | 29.1 (26.1, 31.3) | 31.8 (27.0, 35.0) | 0,200 | 0.4 |
| HDA2.[mg/dL] | 29.2 (26.5, 31.7) | 31.8 (27.2, 35.8) | 0,200 | 0.5 |
| IDAB.[mg/dL] | 3.26 (2.54, 4.02) | 3.48 (2.69, 5.01) | 0,200 | 0.5 |
| V3CH.[mg/dL] | 2.65 (1.85, 2.82) | 3.41 (2.47, 4.29) | 0,200 | 0.5 |
| V3FC.[mg/dL] | 0.93 (0.72, 1.20) | 1.32 (0.84, 1.89) | 0,200 | 0.4 |
| V1PL.[mg/dL] | 3.6 (2.8, 4.9) | 6.2 (3.8, 7.7) | 0,200 | 0.5 |
| V4PL.[mg/dL] | 2.84 (2.68, 3.84) | 3.65 (3.23, 4.36) | 0,200 | 0.5 |
| L3CH.[mg/dL] | 11.0 (9.2, 13.0) | 13.9 (11.4, 16.9) | 0,200 | 0.4 |
| L3FC.[mg/dL] | 3.49 (2.53, 4.42) | 4.05 (2.99, 5.18) | 0,200 | 0.5 |
| L3PL.[mg/dL] | 6.71 (5.86, 7.72) | 8.07 (6.58, 9.15) | 0,200 | 0.5 |
| H2PL.[mg/dL] | 12.06 (10.91, 13.22) | 10.62 (8.83, 13.04) | 0,200 | 0.5 |
| IDPN.[nmol/L] | 59 (46, 73) | 63 (49, 91) | 0,200 | 0.5 |
| ABA1.[-/-] | 0.48 (0.39, 0.56) | 0.52 (0.46, 0.58) | 0,200 | 0.5 |
| Histidine.(rawConc).[mmol/L] | 0.092 (0.084, 0.097) | 0.084 (0.077, 0.092) | 0,200 | 0.4 |
| Valine.(rawConc).[mmol/L] | 0.25 (0.23, 0.27) | 0.25 (0.22, 0.26) | 0,200 | 0.5 |
| Succinic.acid.(rawConc).[mmol/L] |  |  | 0,200 | 0.5 |
| 3-Hydroxybutyric.acid.(rawConc).[mmol/L] | 0.04 (0.03, 0.12) | 0.03 (0.02, 0.04) | 0,200 | 0.4 |
| Acetoacetic.acid.(rawConc).[mmol/L] | 0.007 (0.005, 0.016) | 0.005 (0.004, 0.010) | 0,200 | 0.5 |
| Acetone.(rawConc).[mmol/L] | 0.026 (0.019, 0.054) | 0.020 (0.017, 0.031) | 0,200 | 0.4 |
| Glycerol.(rawConc).[mmol/L] |  |  | 0,200 | 0.5 |
| IDCH.[mg/dL] | 6.8 (6.0, 10.0) | 9.3 (6.1, 13.8) | 0,300 | 0.5 |
| IDFC.[mg/dL] | 2.06 (1.80, 2.68) | 2.74 (1.79, 3.98) | 0,300 | 0.5 |
| V4CH.[mg/dL] | 3.32 (2.85, 4.56) | 4.20 (3.47, 5.12) | 0,300 | 0.5 |
| L1FC.[mg/dL] | 5.63 (4.90, 6.30) | 6.41 (4.39, 7.52) | 0,300 | 0.6 |
| L3AB.[mg/dL] | 6.80 (5.81, 7.93) | 8.28 (6.40, 9.44) | 0,300 | 0.5 |
| H3A1.[mg/dL] | 20.5 (17.4, 23.2) | 24.0 (19.0, 27.2) | 0,300 | 0.5 |
| H1A2.[mg/dL] | 2.63 (2.21, 3.01) | 2.05 (1.39, 2.82) | 0,300 | 0.5 |
| L3PN.[nmol/L] | 124 (106, 144) | 151 (116, 172) | 0,300 | 0.5 |
| 2-Aminobutyric.acid.(rawConc).[mmol/L] | 0.027 (0.005, 0.034) | 0.016 (0.000, 0.027) | 0,300 | 0.5 |
| Glutamine.(rawConc).[mmol/L] | 0.73 (0.68, 0.80) | 0.71 (0.67, 0.78) | 0,300 | 0.5 |
| N,N-Dimethylglycine.(rawConc).[mmol/L] | 0.0060 (0.0035, 0.0070) | 0.0070 (0.0050, 0.0070) | 0,300 | 0.5 |
| Tyrosine.(rawConc).[mmol/L] | 0.053 (0.047, 0.063) | 0.059 (0.056, 0.064) | 0,300 | 0.5 |
| Acetic.acid.(rawConc).[mmol/L] | 0.034 (0.020, 0.051) | 0.026 (0.020, 0.033) | 0,300 | 0.5 |
| V5FC.[mg/dL] | 0.50 (0.38, 0.64) | 0.61 (0.40, 0.81) | 0,400 | 0.6 |
| V5PL.[mg/dL] | 1.28 (1.11, 1.44) | 1.34 (1.21, 1.66) | 0,400 | 0.6 |
| L1TG.[mg/dL] | 3.41 (2.63, 4.58) | 4.06 (3.08, 4.91) | 0,400 | 0.6 |
| L1CH.[mg/dL] | 20 (17, 22) | 22 (16, 25) | 0,400 | 0.6 |
| L1AB.[mg/dL] | 10.0 (8.9, 11.1) | 10.9 (7.7, 13.0) | 0,400 | 0.6 |
| H1TG.[mg/dL] | 2.84 (2.27, 3.50) | 2.46 (2.01, 3.20) | 0,400 | 0.6 |
| H2TG.[mg/dL] | 1.56 (1.35, 1.74) | 1.32 (1.25, 1.64) | 0,400 | 0.6 |
| H3TG.[mg/dL] | 1.56 (1.42, 1.87) | 1.86 (1.47, 2.00) | 0,400 | 0.6 |
| H2CH.[mg/dL] | 7.58 (6.04, 8.52) | 6.91 (5.28, 7.79) | 0,400 | 0.6 |
| H3CH.[mg/dL] | 7.97 (7.28, 9.22) | 9.00 (7.49, 10.36) | 0,400 | 0.6 |
| H1FC.[mg/dL] | 3.38 (2.34, 4.44) | 4.12 (2.42, 4.86) | 0,400 | 0.6 |
| L1PN.[nmol/L] | 182 (161, 202) | 197 (139, 236) | 0,400 | 0.6 |
| Alanine.(rawConc).[mmol/L] | 0.43 (0.40, 0.45) | 0.41 (0.34, 0.44) | 0,400 | 0.6 |
| Threonine.(rawConc).[mmol/L] | 0.13 (0.03, 0.18) | 0.16 (0.11, 0.19) | 0,400 | 0.6 |
| Citric.acid.(rawConc).[mmol/L] | 0.137 (0.115, 0.159) | 0.120 (0.109, 0.141) | 0,400 | 0.6 |
| Glucose.(rawConc).[mmol/L] | 4.54 (4.37, 4.86) | 4.70 (4.63, 4.89) | 0,400 | 0.6 |
| HDCH.[mg/dL] | 44 (37, 56) | 52 (43, 55) | 0,500 | 0.6 |
| L6TG.[mg/dL] | 2.71 (1.69, 2.92) | 2.82 (2.62, 3.31) | 0,500 | 0.6 |
| L2CH.[mg/dL] | 16.9 (15.0, 20.4) | 15.2 (12.1, 19.4) | 0,500 | 0.6 |
| L1PL.[mg/dL] | 11.5 (10.3, 12.4) | 12.3 (9.1, 13.7) | 0,500 | 0.6 |
| L2PL.[mg/dL] | 9.35 (8.55, 11.22) | 8.38 (6.61, 10.67) | 0,500 | 0.6 |
| L2AB.[mg/dL] | 8.77 (8.09, 10.81) | 8.22 (6.56, 10.35) | 0,500 | 0.7 |
| H1PL.[mg/dL] | 21.8 (12.4, 25.0) | 17.3 (11.1, 22.8) | 0,500 | 0.6 |
| H1A1.[mg/dL] | 30 (12, 33) | 22 (16, 29) | 0,500 | 0.7 |
| H2A2.[mg/dL] | 3.07 (2.89, 3.73) | 2.90 (2.26, 3.57) | 0,500 | 0.6 |
| L2PN.[nmol/L] | 159 (147, 196) | 149 (119, 188) | 0,500 | 0.7 |
| Pyruvic.acid.(rawConc).[mmol/L] | 0.05 (0.02, 0.08) | 0.05 (0.04, 0.08) | 0,500 | 0.6 |
| Ca-EDTA.(rawConc).[mmol/L] | | | 0,500 | 0.6 |
| HDTG.[mg/dL] | 8.58 (7.33, 10.01) | 9.53 (7.76, 10.20) | 0,600 | 0.7 |
| L2TG.[mg/dL] | 1.72 (1.43, 2.17) | 1.53 (1.29, 2.31) | 0,600 | 0.7 |
| Trimethylamine-N-oxide.(rawConc).[mmol/L] | 0.018 (0.012, 0.030) | 0.015 (0.011, 0.020) | 0,600 | 0.7 |
| Asparagine.(rawConc).[mmol/L] |  |  | 0,600 | 0.7 |
| Ornithine.(rawConc).[mmol/L] | 0.023 (0.011, 0.045) | 0.029 (0.011, 0.050) | 0,600 | 0.8 |
| Sarcosine.(rawConc).[mmol/L] | 0.0040 (0.0030, 0.0068) | 0.0040 (0.0030, 0.0090) | 0,600 | 0.7 |
| Formic.acid.(rawConc).[mmol/L] | 0.020 (0.014, 0.022) | 0.019 (0.016, 0.023) | 0,600 | 0.8 |
| Dimethylsulfone.(rawConc).[mmol/L] | 0.008 (0.006, 0.009) | 0.007 (0.005, 0.010) | 0,600 | 0.8 |
| HDPL.[mg/dL] | 63 (61, 76) | 68 (59, 77) | 0,700 | 0.8 |
| V5CH.[mg/dL] | 1.15 (1.01, 1.35) | 1.12 (0.93, 1.29) | 0,700 | 0.8 |
| L3TG.[mg/dL] | 1.72 (1.47, 2.05) | 1.63 (1.42, 2.18) | 0,700 | 0.8 |
| H1CH.[mg/dL] | 16.1 (7.7, 19.1) | 14.9 (10.8, 16.8) | 0,700 | 0.8 |
| H3PL.[mg/dL] | 13.59 (11.51, 15.80) | 15.16 (11.37, 16.43) | 0,700 | 0.8 |
| H3A2.[mg/dL] | 5.16 (4.82, 6.10) | 6.21 (4.91, 6.92) | 0,700 | 0.8 |
| Creatine.(rawConc).[mmol/L] | 0.013 (0.009, 0.020) | 0.013 (0.010, 0.019) | 0,700 | 0.8 |
| L5TG.[mg/dL] | 1.38 (0.88, 1.85) | 1.43 (1.18, 1.74) | 0,800 | 0.9 |
| H2FC.[mg/dL] | 1.72 (1.53, 1.99) | 1.78 (1.51, 2.18) | 0,800 | 0.9 |
| Glycine.(rawConc).[mmol/L] | 0.31 (0.28, 0.34) | 0.30 (0.28, 0.34) | 0,800 | 0.9 |
| Proline.(rawConc).[mmol/L] | 0.00 (0.00, 0.21) | 0.00 (0.00, 0.19) | 0,800 | 0.9 |
| 2-Oxoglutaric.acid.(rawConc).[mmol/L] |  |  | 0,800 | >0.9 |
| L4TG.[mg/dL] | 1.51 (0.70, 1.78) | 1.47 (0.90, 1.74) | 0,900 | >0.9 |
| Lysine.(rawConc).[mmol/L] | 0.19 (0.00, 0.22) | 0.19 (0.18, 0.19) | 0,900 | >0.9 |
| Choline.(rawConc).[mmol/L] |  |  | 0,900 | >0.9 |
| LDTG.[mg/dL] | 13.3 (9.9, 15.8) | 12.2 (11.4, 15.3) | >0,9 | >0.9 |
| L2FC.[mg/dL] | 4.78 (4.24, 6.03) | 4.93 (3.70, 6.10) | >0,9 | >0.9 |
| H2A1.[mg/dL] | 15.2 (13.7, 17.9) | 15.7 (12.9, 18.1) | >0,9 | >0.9 |
| Ethanol.(rawConc).[mmol/L] |  |  | >0,9 | >0.9 |
| Glutamic.acid.(rawConc).[mmol/L] | 0.08 (0.05, 0.13) | 0.07 (0.04, 0.13) | >0,9 | >0.9 |
| Methionine.(rawConc).[mmol/L] | 0.046 (0.015, 0.060) | 0.047 (0.020, 0.053) | >0,9 | >0.9 |
| Lactic.acid.(rawConc).[mmol/L] | 2.11 (2.01, 2.45) | 2.26 (1.97, 2.58) | >0,9 | >0.9 |
| K-EDTA.(rawConc).[mmol/L] | 0.02 (0.02, 0.02) | 0.02 (0.02, 0.02) | >0,9 | >0.9 |
| 1 Median (IQR); n (%) | |  |  |  |
| 2 Wilcoxon rank sum test; Pearson’s Chi-squared test; Wilcoxon rank sum exact test; Fisher’s exact test | | | | |
| 3 False discovery rate correction for multiple testing | | | |  |
